# Deciphering the unique cellulose degradation mechanism of the ruminal bacterium *Fibrobacter succinogenes* S85

**DOI:** 10.1038/s41598-019-52675-8

**Published:** 2019-11-12

**Authors:** Mahendra P. Raut, Narciso Couto, Esther Karunakaran, Catherine A. Biggs, Phillip C. Wright

**Affiliations:** 1 The ChELSI Institute, Department of Chemical and Biological Engineering, University of Sheffield, Mappin Street, Sheffield, S1 3JD UK; 2 Centre for Applied Pharmacokinetic Research, University of Manchester, Stopford Building, Oxford Road, Manchester, M13 9PT UK; 3 School of Engineering, Faculty of Science, Agriculture & Engineering, Newcastle University, Newcastle upon Tyne, NE1 7RU UK

**Keywords:** Proteomics, Membranes, Microbiology

## Abstract

*Fibrobacter succinogenes* S85, isolated from the rumen of herbivores, is capable of robust lignocellulose degradation. However, the mechanism by which it achieves this is not fully elucidated. In this study, we have undertaken the most comprehensive quantitative proteomic analysis, to date, of the changes in the cell envelope protein profile of *F. succinogenes* S85 in response to growth on cellulose. Our results indicate that the cell envelope proteome undergoes extensive rearrangements to accommodate the cellulolytic degradation machinery, as well as associated proteins involved in adhesion to cellulose and transport and metabolism of cellulolytic products. Molecular features of the lignocellulolytic enzymes suggest that the Type IX secretion system is involved in the translocation of these enzymes to the cell envelope. Finally, we demonstrate, for the first time, that cyclic-di-GMP may play a role in mediating catabolite repression, thereby facilitating the expression of proteins involved in the adhesion to lignocellulose and subsequent lignocellulose degradation and utilisation. Understanding the fundamental aspects of lignocellulose degradation in *F. succinogenes* will aid the development of advanced lignocellulosic biofuels.

## Introduction

Increasing global energy demand and the continuing depletion of fossil fuels has resulted in an urgent need to establish energy security through the exploration of fuel alternatives such as biofuels. Industrial scale biotechnological production of biofuels such as ethanol and butanol is a reality, but it is not sustainable, as the production process currently utilises food-based feedstocks. Non-food based lignocellulose biomass – comprising cellulose, hemicellulose and lignin – is an emerging sustainable feedstock alternative. The recalcitrant nature of lignocellulose necessitates a two-step process for biofuel production: (i) saccharification for the generation of fermentable sugars (pre-treatment) and (ii) fermentation to biofuels. The primary bottleneck in the production of economically viable lignocellulosic bio-based commodity chemicals is saccharification. Current industrial scale lignocellulosic biofuel generation is expensive, as the production process is heavily dependent upon energy-intensive physical and chemical saccharification steps. With more intensive research efforts, biological saccharification using lignocellulose-degrading microorganisms could be a viable alternative. Consolidated bioprocessing (CBP), i.e. use of native or recombinant microorganisms for both saccharification and fermentation, will be a major breakthrough for the realisation of cost-effective and sustainable lignocellulosic biofuels^1^.

*F. succinogenes* S85 is an efficient lignocellulose degrader isolated from the rumen of herbivores. Intensive investigations over the past three decades indicates that *F. succinogenes* S85 uses an orthogonal lignocellulose degradation system compared to model lignocellulose-degrading microorganisms, as it does not possess either a cellulosome as seen in *Clostridium thermocellum*^2^ or a free cellulolytic enzyme secretion system as seen in *Trichoderma reesei*^3^. Previous studies have indicated that adhesion of cells to cellulose is a crucial process for cellulolysis^4–6^ and a recent study has highlighted the role of extracellular vesicles in cellulose degradation^7^. However, the enigmatic cellulose degradation mechanism employed by *F. succinogenes* S85 is not fully understood. A deeper understanding of the lignocellulose degradation mechanism in *F. succinogenes* S85 will allow the use of this microorganism to accelerate CBP development.

The complete genome of *F. succinogenes* S85 was sequenced in 2011^5^. Although 50% of the genome could not be annotated as encoding proteins in known functional categories, the genome revealed the presence of a high number of genes encoding glycoside hydrolase (GH) domain-bearing proteins. GH domains are responsible for cellulolytic activity. Efforts have been made to heterologously express individual GH domain bearing proteins for cellulolysis^6,8,9^. It was found that the cellulolysis was much lower than that seen in *F. succinogenes* S85. This indicates that a synergistic mechanism of cellulolytic degradation is utilised by *F. succinogenes* S85. Indeed when a combination of multiple GH proteins were heterologously expressed, the cellulolysis improved compared to the heterologous expression of individual GH proteins^10^. However, the overall cellulolytic potential of the recombinant bacterium was still lower than that of *F. succinogenes* S85. This suggests that further elucidation of the synergy between GH proteins in *F. succinogenes* S85 is required.

Although the genome of *F. succinogenes* S85 has been widely available since 2011, studies utilising post-genomic era tools such as transcriptomics and proteomics to uncover the physiology of this bacterium have been scarce. Neumann *et al*.^11^ employed transcriptomics to compare global expression of genes in *F. succinogenes* S85 when grown on glucose, cellobiose and cellulose. They found distinct patterns of gene expression particularly for genes encoding cellulases and hemicellulases when cells were grown on different carbon sources. Our research team was the first to employ cutting edge, gel-free semi-quantitative proteomics techniques to compare the differences in cell envelope proteome in *F. succinogenes* S85 when grown using glucose or cellulose as sole carbon sources^12^. We demonstrated that when growing as biofilms on cellulose, as hypothesised in previous studies, the cellulose degradation machinery is indeed localised in the cell envelope of *F. succinogenes* S85 and we identified important accessory features of the lignocellulose degradation process. However, several crucial mechanistic questions remain unanswered; What are the key proteins involved in lignocellulose degradation? How are these proteins organised? What are the processes that *F. succinogenes* S85 utilises to achieve complete degradation and utilisation of lignocellulose?

Therefore, in this study, in order to further functionally elucidate the processes and key elements involved in the lignocellulose degradation mechanism in *F. succinogenes* S85, we have for the first time, combined biotin-neutravidin affinity-based cell envelope protein enrichment with quantitative proteomics using iTRAQ. Enzymatic assays, lipopolysaccharide (LPS) analysis, scanning electron microscopy (SEM) analysis and cyclic-di-guanidine monophosphate (GMP) quantification were used to functionally validate the iTRAQ results.

## Results

The experimental design used in this work is shown in Fig. 1 in Supplementary File 1. Our approach allowed us to identify and quantify 1043 proteins with at least 2 unique peptides at a false discovery rate (FDR) < 1% (Supplementary File 2). From the quantified proteins, 464 proteins were differentially abundant between cellulose- and glucose-grown cells. Of these 464 proteins, 273 proteins were predicted to be of non-cytoplasmic or of unknown localisation and 191 proteins were predicted to be cytoplasmic (Supplementary File 3). Further subcellular localisation analysis predicted that these 273 non-cytoplasmic proteins were made up of 10 extracellular proteins, 18 outer membrane proteins, 14 periplasmic proteins, 41 inner membrane proteins and 190 non-cytoplasmic proteins with unknown location (Supplementary File 3).

### Regulation of lignocellulose degradation enzymes

At least 31 genes encoding cellulases have been predicted in the *F. succinogenes* S85 genome^5^. Of these, we quantified 18 predicted cellulases in the cell envelope proteome, 6 of which were not significantly regulated (FSU_2070, FSU_2534, FSU_0451, FSU_0810, FSU_2558, FSU_1947). From the remaining 12 predicted cellulases, 10 were observed to be up-regulated, whilst 2 were found to be down-regulated in the cell envelope proteome of cellulose-grown cells. The cellulolytic activity of 90% of the predicted cellulases that were significantly up-regulated in the cell envelope of cellulose-grown cells has been previously confirmed experimentally (Table 1). The cellulolytic activity of the two predicted cellulases that were found to be down-regulated has not been confirmed experimentally. Overall, our results indicate that the expression of these cellulases are regulated in response to the presence of cellulose and functionally confirm the role of the 10 up-regulated cellulases in the degradation of microcrystalline cellulose by *F. succinogenes* S85.

**Table 1:** Major lignocellulose degradation enzymes differentially abundant in the cell envelope proteome during cellulose degradation.

*Protein ID*	*Gene ID*	*Protein names*	*Fold change*^*§*^	*P-value*	Carbohydrate active domains*	*Signal peptide (Residues)*	*BTD (Residues)*	T9SS signal ** (Residues)	*Predicted activity*	*Confirmed activity*	*CBM binding specificity*
***Cellulases***											
C9RQE4	FSU_0382	Cellulase	1.46	****	CBM30,CBM30,CBM11,GH51	(1–24)	na	na	β-glucanase	Cellulase^9^	Cellulose single chains
A7UG57	FSU_0809	Glycoside hydrolase 9	−1.20	***	GH9	(1–19)	na	na	Cellulase	—	—
C9RNF0	FSU_1228	Cellulase	1.47	**	GH5	(1–22)	na	Yes	Cellulase	Cellulase^5^	—
C9RQJ1	FSU_1685	Cellulase	1.48	****	GH5	(1–21)	na	TIGR04183 (677–742)	Cellulase	Cellulase^5^ Multi-protein complex^7^	—
C9RR37	FSU_1893	Endoglucanase	1.36	***	GH45	(1–19)	na	na	Cellulase	Cellulase^27^	—
C9RRD4	FSU_2005	Cellulase	1.38	**	GH5	(1–20)	na	na	Cellulase	Cellulase^5^	—
A7UG68	FSU_2303	Glycoside hydrolase 8	1.33	****	GH8	na	BTD type II (673–752)^10^	Yes	Endoglucanase	Cellulase^10^ Multi-protein complex^7^	—
A7UG67	FSU_2361	Endoglucanase	1.64	**	GH9 (CBM48)	(1–27)	BTD (578–620)^10^	na	β-glucanase	Cellulase^47^	—
D9S4N9	FSU_2362	Endoglucanase	1.81	***	GH9 (CBM48)	(1–24)	BTD (620–665)^47^	na	Cellulase	Multi-protein complex^7^	—
P14250	FSU_2772	Endoglucanase 3 (Cellulase 3)	1.83	****	GH5 (CBM11)	(1–26)	na	na	Endoglucanase	Cellulase^48^	Cellulose single chains
A7UG69	FSU_2914	Cellulase	1.93	****	GH5 (CBM11)	(1–21)	BTD type II (821– 910)^10^	TIGR04183 (841–910)	Cellulase	Cellulase^10^ Multi-protein complex^7^	Cellulose single chains
C9RMD2	FSU_3149	Endo-1,4-β-glucanase	−2.11	***	GH8	(1–21)	na	TIGR04183 (420–478)	β-glucanase	Cellulase;***^27^	—
***Endo- and exo- hemicellulases***											
P35811	FSU_0777	Endo-1,4-β-xylanase C (Xylanase C)	1.68	*	GH11,GH11	(1–26)	FPm-1 (538–608)^13^ BTD^5,49^	Yes	Xylanase	Xylanase^49^	—
A7UG63	FSU_2012	Chitinase	−1.30	***	GH18 CBP-9	(1–20)	na	na	Chitinase	Cellulose binding^50^	—
C9RS20	FSU_2263	Endo-1,4-β-xylanase	1.53	*	GH43,CBM6,CBM6,CBMnc	(1–23)	FPm-1 (670–740)^13^ BTD^5^	Yes	β-xylosidase	—	Hemicellulose single chain
C9RS21	FSU_2264	Endo-1,4-β-xylanase	1.95	***	GH43,CBM6,CBM6	(1–23)	FPm-1 (664-730)^13^ BTD^5^	Yes	β-xylosidase	—	Hemicellulose single chain^27^
C9RS26	FSU_2269	Xylanase/xylosidase	1.41	**	GH43,CBM6,CBMnc	(1–24)	FPm-1 (708-778)^13^ BTD^5^	Yes	β-xylosidase	Arabinoxylanase^5^	Hemicellulose single chain
D9S442	FSU_2274	Xylanase/xylosidase	1.73	**	GH43,CBM6,CBM6	(1–22)	FPm-1 (623–688)^13^ BTD^5^	Yes	β-xylosidase	—	Hemicellulose single chain
C9RS45	FSU_2288	β-galactosidase	1.41	***	GH2,CBMnc	(1–24)	FPm-1 (1098–1165)^13^ BTD^5^	TIGR04183 (1088–1165)	β-galactosidase	—	
Q9F4L0	FSU_2292	β-xylanase	1.35	**	GH10,CBM6,CBMnc	(1–23)	FPm-1 (554–623)^13^ BTD^5^	Yes	Xylanase	—	Hemicellulose single chain^51^
Q9F108; D9S458	FSU_2293 or FSU_2294	β-xylanase	1.66	***	GH10,CBM6,CBMnc	(1–24)	FPm-1 (2293: 547–616, 2294: 519–588)^13^ BTD^5^	Yes	Xylanase	Xylanase^51^	Hemicellulose single chain
***Other GH domain containing enzymes***											
C9RP13	FSU_0162	Cellobiose/cellodextrin phosphorylase	1.42	****	GH94	na	na	na	cellodextrin-phosphorylase	—	—
D9S524	FSU_0196	Mannanase	1.27	****	GH5	na	na	na	mannanase	β-mannanase	—
D9S5W9	FSU_0369	1,4-α-glucan branching enzyme	1.38	****	GH13	na	na	na	1,4-α-glucan branching	—	—
D9S9L7	FSU_1169	Glycosyl hydrolase 57	−1.28	*	GH57	na	na	na	α-amylase	α-amylase	—
C9RNM3	FSU_1304	4-α-glucanotransferase	1.36	****	GH77	na	na	na	4-α-glucano-transferase	—	—
C9RS30	FSU_2272	α-galactosidase	1.45	**	GH27,CBM6	(1–30)	FPm-1 (565–630)^13^ BTD^5^	Yes	α-galactosidase	α-galactosidase	Hemicellulose single chain
C9RKA3	FSU_2795	Xylanase-like protein	−1.41	****	GH30	(1–18)	na	TIGR04183 (660–714)	Xylanase	—	—
C9RLJ5	FSU_2986	Glycosyl hydrolase 16	−1.68	*	GH16	(1–20)	BTD^5^	Yes	Xylanase	—	—
C9RN35	FSU_3272	Conserved domain protein	1.40	****	GH116	na	na	na	Glucosyl-ceramidase	—	—

*Carbohydrate active domains are annotated based on CAZy database^52^. Domains in brackets are annotated in Uniprot and NCBI Conserved Domain Database^53^.

**Sequence alignments are provided in Supplementary Information.

***Activity checked only against 1, 3 barley glucan, not microcrystalline cellulose.

na - not annotated.

*p*-value range denoted as *0.05–0.01, **0.01–0.001, ***0.001–0.0001 and ****<0.0001.

^§^Fold changes of the differentially abundant proteins in cellulose-grown cells versus glucose-grown cells were calculated with 95% significance^45^. Please see Supplementary File 2.

In addition to cellulases, 8 endo- and exo- hemicellulases as well as 6 other GH family proteins were up-regulated (except chitinase FSU_2012, GH 57 family protein FSU_1169, Xylanase like protein FSU_2795, GH 16 family protein FSU_2986) in the envelope of cellulose-grown cells (Table 1). Increased expression of hemicellulases and other GH domain proteins in cells grown in the presence of cellulose as the sole carbon source confirms that the expression of these proteins, as well as the cellulases, are controlled at a global level through catabolite repression.

More crucially, our results highlight the localisation of these cellulases and other polysaccharide degrading enzymes on the cell envelope. The extra-cytoplasmic localisation of these enzymes is supported by the presence of a characterised N-terminal signal peptide in a majority of these proteins (Table 1). However, a typical cell surface anchoring domain has not previously been demonstrated for these enzymes. Nevertheless, the presence of a positively charged, highly basic domain (pI > 9.0) at the C-terminus of most of these enzymes, designated as basic terminal domain (BTD) or Fibrobacter paralogous module 1 (Fpm-1) domain^13,14^ has been discussed previously as a cell-surface anchoring domain of unknown mechanism (Table 1). We noticed that the BTD (residues 821–910) at the C-terminus of a highly up-regulated cellulase (FSU_2914) was recently annotated as a Type 9 secretion signal (T9ss; TIGRFAM04183 Por secretion tail). We also found a similar overlap between BTD/Fpm-1 domain and T9ss domain in another protein, β-galactosidase (FSU_2288). Similar to BTD and Fpm-1 domains, T9ss domains are known to be highly basic in nature. Therefore, we propose that the BTD/Fpm-1 domain in *F. succinogenes* S85 is a T9ss signal. Five proteins in our results contain the T9ss signal (TIGRFAM04183) (Table 1). By sequence homology (data not shown) we further identified 11 proteins that might possess T9ss signal domains (TIGRFAM04183) (Table 1). Our proposal that the BTD/Fpm-1 domain is a T9ss signal is further supported by our observation that the peptides identified in the mature protein by mass spectrometry do not overlap with the BTD/Fpm-1 domain, suggesting that they have undergone cleavage during attachment of the mature protein to the outer membrane (Supplementary File 1, Supplementary Note). Based on our results, we suggest that the lignocellulose degradation machinery in *F. succinogenes* S85 is transported through the cell envelope and is covalently attached to the outer membrane by the T9ss mechanism. In order to avoid confusion in the literature with regard to terminology of the C-terminal domain, we suggest that henceforth the BTD/Fpm-1 domain be referred to as the C-terminal domain (CTD) to streamline with the terminology used to refer to this domain in T9ss.

Although localisation of the degradative enzymes on the cell surface favours degradation of insoluble lignocellulose, the glycosidic bonds that are cleaved by these enzymes are often not easily accessible on the insoluble substrate. Carbohydrate binding modules (CBMs) found on such degradative enzymes are known to play a crucial role in enhancing lignocellulose degradation by bringing the glycosidic bonds of the substrate within close proximity to the active site of these enzymes^15^. Accordingly, the cellulases found up-regulated in the cell envelope of cellulose-grown cells contain either CBM11 or CBM30 domains or both (Table 1), which are known to effectively bind to cellulose^5^. The association of hemicellulases up-regulated in cellulose grown cells, with CBM6 domains - known to bind hemicellulose^16^ - is also evident from our results (Table 1). Although some of the up-regulated enzymes do not contain CBM domains, their presence cannot be ruled out, as several CBMs are yet to be discovered^17^. Cumulatively, our results demonstrate that the lignocellulose degradative enzymes are localised on the cell surface of *F. succinogenes* S85, that they mediate binding to polysaccharides and that they may be regulated *via* catabolite repression.

### Proposed model of cell surface/envelope associated multi-protein complexes in lignocellulose adhesion and degradation

We quantified 15 TPR domain proteins, 13 OmpA proteins, 6 fibroslime proteins and 2 pili proteins in the cell-envelope proteome. Of these, 7 TPR domain proteins, 8 OmpA proteins and 4 fibroslime proteins were found to be differentially regulated in cellulose-grown cells, whilst the rest were present but not differentially regulated. Most of these up-regulated proteins possess a N-terminal secretion signal, confirming their extra-cytoplasmic localisation (Table 2).

**Table 2:** Differential abundance of proteins proposed to be present in multi-protein complexes on the cell surface of *F. succinogenes* S85 during cellulose degradation

*Protein ID*	*Gene ID*	*Protein name*	*Fold change*^*§*^	*P-value*	*Signal peptide (Residues)*	*COG motif*	*Domains*	*Confirmed activity*
***Tetratricopeptide domain protein***								
D9S777	FSU_0603	Putative lipoprotein	−1.37	**	na	TPR	—	—
A7UG62	FSU_2397	TPR domain protein	1.46	****	(1–23)	TPR	—	Multi-protein complex^4,7^
A7UG58	FSU_2398	TPR domain protein	1.2	****	(1–20)	TPR	—	^4^
C9RPX7	FSU_0345	Putative lipoprotein	1.19	*	na	TPR	—	—
C9RJ09	FSU_0431	Tetratricopeptide repeat protein	−1.39	***	na	TPR	—	—
C9RKI3	FSU_0711	Tetratricopeptide repeat protein	−1.45	****	(1–29)	TPR	—	—
C9RRR7	FSU_2147	Tetratricopeptide repeat protein	−1.84	****	(1–22)	TPR	—	—
***Fibroslime proteins***								
D9SB51	FSU_1795	Conserved domain protein	1.71	****	na	PA14	Fibroslime	—
A7UG66	FSU_2502	Fibro-slime domain protein	1.66	****	(1–32)	PA14	Fibroslime	^5,54^Multi-protein complex^7^
D9S827	FSU_0792	Uncharacterized protein	1.43	****	na	PA14	Fibroslime	—
C9RR85	FSU_1953	Conserved domain protein	1.34	****	(1–20)	PA14	Fibroslime	—
***OmpA domains***								
A7UG61	FSU_2396	OmpA family protein	1.43	****	(1–28)	OmpA	—	Multi-protein complex^4,7^
C9RLT0	FSU_3077	OmpA family protein	1.3	****	(1–19)	OmpA	—	^4^Multi-protein complex^7^
C9RQ78	FSU_1609	OmpA family protein	1.25	**	na	OmpA	—	—
C9RP29	FSU_0180	OmpA family protein	−1.2	****	(1–17)	OmpA	—	^4^
C9RJU7	FSU_0604	Peptidoglycan-associated lipoprotein	−1.28	****	na	OmpA	—	—
C9RM27	FSU_1003	OmpA family protein	−1.59	**	(1–17)	OmpA	—	—
D9S4Y4	FSU_0151	OmpA family protein	−1.69	****	na	OmpA	—	—
C9RNK7	FSU_1288	OmpA family protein	−3.78	***	(1–21)	OmpA	—	—
***Pilin proteins***								
C9RN03	FSU_1212	Pilin domain protein	1.26	*	na	N-methyl-site	Adhesion	—
A7UG50	FSU_2567	Type IV pilin	1.17	***	na	N-methyl-site	Adhesion	Absent in cellulolysis deficient mutants^4^

na - not annotated.

*p*-value range denoted as *0.05–0.01, **0.01–0.001, ***0.001–0.0001 and ****<0.0001.

^§^Fold changes of the differentially abundant proteins in cellulose-grown cells versus glucose-grown cells were calculated with 95% significance^45^. Please see Supplementary File 2.

**Figure 1:**
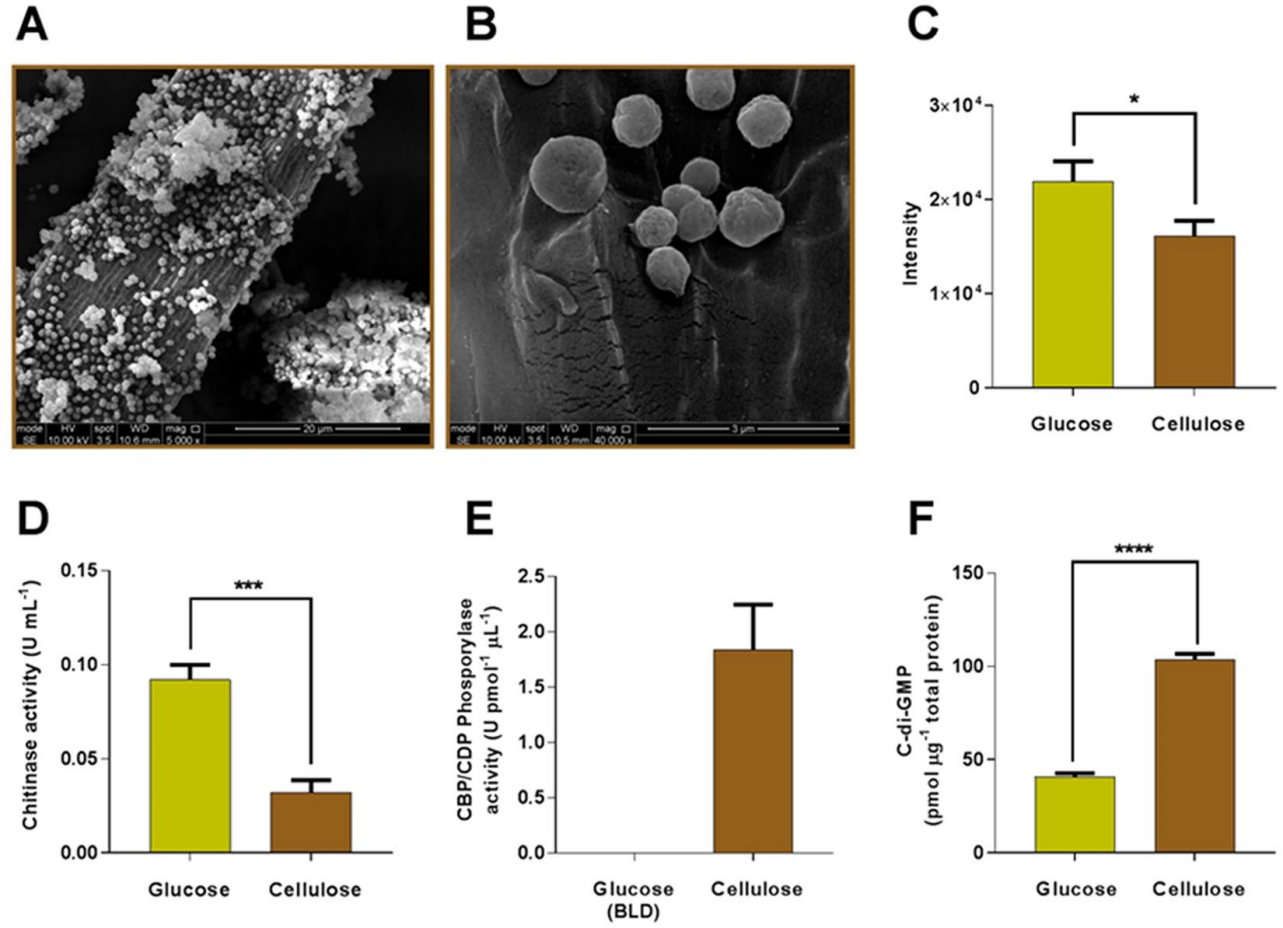
Changes in morphology, enzymatic and metabolite profile of *F. succinogenes* S85 during growth on cellulose. Scanning electron microscopy of *F. succinogenes* S85 cells attached to microcrystalline cellulose. Images showed adherence of cells to microcrystalline cellulose particles. Parallel grooves and pits have been left behind in places where cells have detached from the cellulose particle (**A and B**). Scanning densitometry of O-antigen carbohydrate moiety of LPS obtained from *F. succinogenes* S85 cells grown in glucose and cellulose substrate. Results are an average of three biological replicates (**C**). Chitinase activity of *F. succinogenes* S85 on cellulose vs glucose-grown cells. Results are an average of two biological replicates and two technical replicates (**D**). Cellobiose/cellodextrin phosphorylase activity of *F. succinogenes* S85 on cellulose vs glucose-grown cells. Results are an average of three biological replicates and three technical replicates. In glucose cellobiose/cellodextrin phosphorylase activity was below the limit of detection (BLD) (**E**). Quantification of cyclic-di-GMP (c-di-GMP) in response to cellulose and glucose. Results are an average of two biological and two technical replicates (**F**). Error bars indicate standard deviation and stars indicate the level of significance between conditions as determined using two-tailed Student’s *t* test at 95% confidence. *p*-value less than 0.05 and greater than 0.01 is represented by *, *p*-value less than 0.01 but greater that 0.001 is represented by **, *p*-value less than 0.001 but greater than 0.0001 is represented by *** and *p*-value lesser than 0.0001 is represented by ****.

Our observations lead us to suggest that the up-regulated TPR domain proteins on the cell surface bring together the degradative enzymes and fibroslime proteins in a potentially cellulolytic multi-protein complex, which is anchored to the peptidoglycan *via* the up-regulated outer membrane spanning OmpA family proteins. These proposed multi-protein complexes mediate adhesion of *F. succinogenes* S85 to cellulose (Fig. 1A,B) and subsequent cellulose degradation. The up-regulation of these proteins in cellulose-grown cells further indicates that the expression of the corresponding genes is controlled *via* catabolite repression.

Our results also indicate that the formation of these multi-protein complexes in the outer membrane of cellulose-grown cells is correlated with extensive regulation of proteins involved in trafficking and localisation of cell envelope components. *F. succinogenes* S85 is a Gram negative organism, the cell envelope of which is composed of an outer membrane and an inner membrane separated by a periplasmic region containing a peptidoglycan layer. The outer membrane and the inner membrane are typically composed of a phospholipid bilayer interspersed with lipoproteins. In our results, we observed that the members of the LolABCDE and the Sur/BamABCDE pathways involved in sorting lipoproteins to the outer membrane^18^ were down-regulated in the cell envelope of the cellulose-grown cells (Table 3). In agreement with this, 40 out the 49 putative lipoproteins we quantified in our results were down-regulated (Supplementary File 3). Most of these putative lipoproteins contain a N-terminal secretion signal confirming their extra-cytoplasmic localisation.

**Table 3:** Differential abundance of proteins involved in cell-envelope biogenesis.

*Protein ID*	*Gene ID*	*Protein name*	*Fold change*^*§*^	*P-value*	*Sub-cellular location*	*Signal peptide (Residues)*	*COG motif*
***Outer membrane proteins and lipoproteins biogenesis***							
C9RJJ1	FSU_2655	Outer membrane protein, OmpH family	−2.05	****	Unknown (Multi)	(1–20)	OmpH/Skp
C9RPM2	FSU_0239	PPIC-type PPIASE domain protein	−4.02	****	Unknown (Multi)	(1–23)	Rotamase, SurA
D9S491	FSU_0013	Peptidylprolyl isomerase	−1.38	****	Outermembrane	na	Rotamase, SurA
C9RLW8	FSU_0941	Peptidylprolyl isomerase	−3.42	****	Periplasmic membrane	(1–20)	Rotamase, SurA
D9S652	FSU_2654	Outer membrane protein, OMP85 family	−1.37	****	Outermembrane	na	OMP85/BamA
C9RJE0	FSU_2598	Uncharacterized protein	−2.73	**	Unknown	(1–22)	LolA, LolA_like
***Lipopolysaccharides and phospholipid biogenesis***							
C9RMR9	FSU_1121	Lipid-A-disaccharide synthetase	1.45	**	Unknown	na	LpxB
C9RRK0	FSU_2077	Tyrosine-protein kinase	−1.20	**	Cytoplasmic membrane	na	Wzz
C9RL67	FSU_0817	O-antigen modification glycosyltransferase	−1.20	*	Cytoplasmic membrane	na	Glycosyltransferase
C9RQT0	FSU_1784	Lipoprotein	−3.35	****	Unknown	(1–21)	LpoB, LptE
C9RP30	FSU_0181	Tyrosine-protein kinase	−1.80	****	Cytoplasmic membrane	na	Wzz
D9S4D5	FSU_0064	1-acyl-sn-glycerol-3-phosphate acyltransferase	−1.16	*	Unknown	na	Acyltransferase, Plsc, LPLATs
C9RPR3	FSU_0280	Uncharacterized protein	−1.37	***	Unknown	(1–21)	OMP/PagP_b-brl
A7UG44	FSU_0230	Lipoprotein	−1.15	****	Unknown	(1–20)	OMP/PagP_b-brl
D9SBZ4	FSU_2141	Mce-like protein	−1.20	***	Unknown	na	Mce/MlaD
C9RMP7	FSU_1094	Lipoprotein	−3.68	****	Unknown	(1–19)	MlaC/ttg2D
C9RK73	FSU_2762	Mce-like protein	−1.68	****	Transmembrane	na	Mce/MlaD
C9RM87	FSU_1068	Lipoprotein	−1.31	*	Unknown	na	MlaD
***Peptidoglycan biogenesis and cell division***							
C9RKC5	FSU_2817	Lipoprotein	−1.40	**	Unknown	(1–21)	Carboxypeptidase regulatory-like domain
C9RMP1	FSU_1088	Lipoprotein	−2.59	**	Unknown	(1–23)	Carboxypeptidase regulatory-like domain
C9RRQ4	FSU_2134	Lipoprotein	−1.33	***	Unknown	(1–22)	Carboxypeptidase regulatory-like domain
C9RJW9	FSU_0627	Lipoprotein	−1.15	**	Unknown	na	Carboxypeptidase regulatory-like domain
C9RKE2	FSU_0669	Peptidase, M23/M37 family	−2.65	****	Unknown	(1–22)	Peptidase, M23
C9RKS7	FSU_2836	Uncharacterized protein	1.74	**	Cytoplasmic membrane	na	Peptidase_M23
C9RPX0	FSU_0338	Penicillin binding transpeptidase domain protein	−1.36	**	Unknown	na	DD Transpeptidase
C9RJL9	FSU_0520	Uncharacterized protein	−1.28	**	Cytoplasmic membrane	na	PknB
C9RPF5	FSU_1461	Endolyticmureintransglycosylase	−1.18	**	Transmembrane	na	MltGYceG
C9RQT0	FSU_1784	Lipoprotein	−3.35	****	Unknown	(1–21)	LpoB, LptE
C9RL98	FSU_0847	LysM domain protein	−2.34	**	Unknown (Multi)	(1–21)	LysM
C9RIJ7	FSU_2382	Lipoprotein	−1.60	***	Unknown	na	SPOR
C9RIS5	FSU_2476	Endolytic peptidoglycan transglycosylase	−2.09	*	Unknown	na	RlpA
D9S4A7	FSU_0030	ATP-dependent zinc metalloprotease	−1.32	****	Cytoplasmic membrane	na	FtsH
C9RM12	FSU_0987	Cell division protein	−1.26	****	Unknown (Multi)	na	FtsZ_C
C9RQL6	FSU_1711	Lipoprotein	2.09	**	Unknown	na	Spc7
C9RJR4	FSU_0566	Lipoprotein	−1.50	*	Unknown	na	TusA

na - not annotated.

*p*-value range denoted as *0.05–0.01, **0.01–0.001, ***0.001–0.0001 and ****<0.0001.

^§^Fold changes of the differentially abundant proteins in cellulose-grown cells versus glucose-grown cells were calculated with 95% significance^45^. Please see Supplementary File 2.

In addition to lipoproteins, we observed differential abundance of proteins that are involved in the assembly of the outer membrane lipids. In Gram negative bacteria such as *F. succinogenes* S85, the outer membrane is asymmetrical since the composition of the outer leaflet is composed both of phospholipids and lipopolysaccharides (LPS)^18^. The LPS is made up of a lipid A molecule attached to an O-antigen polysaccharide. We observed an up-regulation of the putative protein LpxB involved in Lipid A synthesis, suggesting an increase in the concentration of lipid A in the outer membrane. In agreement with this, the members of the MlaABCDE involved in maintaining the asymmetry of the outer membrane by increasing phospholipid turnover, was down-regulated in cellulose-grown cells. However, the results suggest that the increase in lipid A moieties in the outer membrane is not correlated with O-antigen synthesis, as proteins involved in O-antigen synthesis and trafficking were down-regulated. This suggests that during cellulose degradation, the LPS molecules have reduced concentration of O-antigen on the cell surface (Table 3). We confirmed these observations by comparing the extracted LPS from cells grown on glucose and cellulose. The LPS extracts were separated by denaturing polyacrylamide gel electrophoresis and the O-antigen was stained using the ProQ Emerald 300 polysaccharide stain (Supplementary File 1, Fig. 2). A densitometric analysis confirms the reduction of O-antigens on the LPS of cells during cellulose degradation (Fig. 1C). The reduction of polysaccharide moieties on the cell surface during cellulose degradation agrees with our previously published observations using Fourier Transform Infrared (FTIR) spectroscopy^12^.

In addition to the regulation of lipoprotein and membrane lipids, our results suggest that peptidoglycan synthesis and turnover may be down-regulated in cells grown on cellulose. Based on the presence of appropriate COG motifs, proteins linked to peptidoglycan synthesis and turnover were identified. All of these proteins are down-regulated in the envelope of cells involved in cellulose degradation (Table 3). This observation led us to hypothesise that the observed down-regulation of chitinase (Table 1) may be linked to decreased peptidoglycan turnover, given the structural similarities between peptidoglycan and chitin. The down-regulation of chitinase activity, specifically the endo-β-N-acetylglucosaminidase activity in the envelope of cellulose-grown cells was confirmed enzymatically (Fig. 1D).

In the light of our results, it may be justified to conclude that potentially cellulolytic multi-protein complexes form at the surface of *F. succinogenes* S85 during growth on cellulose. Such multi-protein complexes facilitate the synergistic action of degradative enzymes, thereby enhancing the degradation of lignocellulose. The simultaneous re-arrangements of multiple cell envelope components indicate that the cell envelope of *F. succinogenes* S85 is geared predominantly towards cellulose degradation and utilisation during growth on cellulose.

### Transport of cellulose degradation products

The concerted effort of the cellulases result in the production of cellodextrins, the products of cellulose degradation. Previous observations demonstrate that, during cellulose degradation by *F. succinogenes* S85, cellodextrins do not accumulate in the extracellular medium^19^. This suggests that *F. succinogenes* S85 possesses extensive protein machinery for efficient transport and utilisation of cellodextrins. Indeed 8% of the whole genome sequence of *F. succinogenes* S85 is predicted to encode proteins involved in cellodextrin transport and utilisation^5^. However, the mechanism of cellodextrin transport remains unknown.

In our results, 11 proteins putatively involved in transport of macromolecules were up-regulated in the cell envelope of cellulose-grown cells (Table 4). Of particular interest is the up-regulation of a gene cluster (FSU_2400 to FSU_2403), predicted to be an operon^20^, encoding proteins with TonB/ExbB/ExbD domains. In Gram negative bacteria, such as *F. succinogenes* S85, the TonB/ExbB/ExbD protein complex is known to facilitate active transport of charged molecules or molecules larger than ~600 Da *via* a TonB dependent outer membrane β-barrel protein. The role of TonB/ExbB/ExbD complex in conjunction with MalA, a β-barrel outer membrane protein in the import of maltodextrins (degradation products of starch) has been experimentally demonstrated in the Gram negative bacterium, *Caulobacter crescentus*. This synergy between MalA and TonB/ExbB/ExbD protein complex was not only essential for uptake of maltotetrose but also increased the uptake of the monomer, maltose, by tenfold^21^. Although we were unable to identify MalA-like protein, based on our observation of the up-regulation of the TonB/ExbB/ExbD complex, we hypothesise that *F. succinogenes* S85 utilises TonB dependent mechanism for the active transport of cellodextrins across the outer membrane.

**Table 4:** Differential abundance of predicted transporters and proteins involved in cyclic-di-GMP synthesis during cellulose degradation.

*Protein ID*	*Gene ID*	*Protein name*	*Fold change*^§^	*p-value*	*Signal peptide (Residues)*	*COG motif*	*Predicted activity*	*Sequence homology*
***ATP binding cassette (ABC) transporters***								
C9RQJ3	FSU_1687	Oligopeptide/dipeptide ABC transporter	1.32	****	na	ABC	Cellobiose/cellodextrin import	40.9% to (TM_0027, TM_1219) and cbtF^22,23,55^
C9RLQ8	FSU_3055	ABC transporter	1.21	*	na	ABC	—	TM_1028 31.8%^56^
C9RQJ4	FSU_1688	Oligopeptide/dipeptide ABC transporter	1.15	**	na	ABC	—	TM_1220 (36%) and cbtD^23,55,57^
***Solute binding protein****(****SBP****)*								
D9S9B3	FSU_1047	Extracellular solute-binding protein	1.25	****	na	SBP	Cellobiose/cellodextrin import	31.3% to (cbpB) A3DE73^58^
C9RLA2	FSU_0851	Extracellular solute-binding protein, family 5	1.19	**	na	SBP	Cellobiose/cellodextrin import	35.3% to cbtA^22^
D9S5U2	FSU_0342	Extracellular solute-binding protein, family 3	1.11	*	na	SBP	—	—
C9RLS4	FSU_3071	Periplasmic amino acid binding	−1.58	****	(1–22)	SBP	—	—
C9RJQ0	FSU_0552	Periplasmic sulfate binding	−3.32	****	(1–23)	SBP	—	—
***Others***								
C9RIL6	FSU_2403	TonB family protein	1.22	***	na	TonB	—	—
A7UG46	FSU_1029	Membrane protein	1.61	****	(1–21)	Porin	Multi-protein complex^7^	—
C9RIL3	FSU_2400	MotA/TolQ/ExbB proton channel family protein	1.50	****	na	ExbB/MotA	—	—
D9S5A5	FSU_0286	Fimbriae-associated domain protein	−1.16	****	na	ExbB/MotA	—	—
C9RJ13	FSU_0435	MotA/TolQ/ExbB proton channel family protein	−1.23	****	(1–50)	ExbB/MotA	—	—
C9RIL5	FSU_2402	Membrane protein	1.73	****	na	ExbD	—	—
A7UG36	FSU_2401	Membrane protein	1.46	****	na	ExbD	—	—
***Cyclic di GMP synthesis***								
C9RKL7	FSU_0748	Response regulator	1.78	**	na	GGDEF, EAL, Response regulator	—	—
A7UG35	FSU_0222	Diguanylate cyclase (GGDEF) domain protein	1.49	*	na	GGDEF	—	—
C9RK09	FSU_2692	Diguanylate cyclase (GGDEF) domain protein	−1.47	*	na	GGDEF, GAF,GAF2	—	—

na - not annotated

*p*-value range denoted as *0.05–0.01, **0.01–0.001, ***0.001–0.0001 and ****<0.0001.

^§^Fold changes of the differentially abundant proteins in cellulose-grown cells versus glucose-grown cells were calculated with 95% significance^45^. Please see Supplementary File 2.

The observed up-regulation of cellobiose/cellodextrin phosphorylase (FSU_0162), predicted to be localised in the inner membrane of cells grown on cellulose (Table 1), was functionally confirmed experimentally using a whole cell-based enzymatic assay (Fig. 1E). This provides insight into the mechanism employed by *F. succinogenes* S85 for the utilisation of cellulose degradation products. Cellobiose/cellodextrin phosphorylase processes cellodextrins into glucose-1-phosphate and smaller chains of cellodextrins, such as cellobiose. The observed up-regulation of 3 ABC transporters and their cognate solute binding proteins (Table 4) may facilitate subsequent transport of glucose-1-phosphate and cellobiose across the inner membrane. Specifically, FSU_1047 and FSU_0851 demonstrate sequence similarities with experimentally validated solute binding proteins involved in transport of cellobiose across the inner membrane in *Thermotoga maritima* and *Pyrococcus furiosus*, which are known to degrade cellulose^22,23^. We suggest that *F. succinogenes* S85 utilises the ABC transporters and solute binding proteins found to be up-regulated in our study for the transport of cellulose degradation products across the inner membrane.

### Role of cyclic-di-GMP in cellulose degradation

In our results, we observed that growth on cellulose elicited the differential abundance of three proteins bearing GGDEF domains (Table 4). GGDEF domain bearing proteins are known to be involved in the synthesis of cyclic-di-GMP, a ubiquitous second messenger molecule, in several Gram negative bacteria. During growth on cellulose, two inner membrane associated proteins bearing the GGDEF domains were significantly up-regulated. The genes encoding these proteins are not predicted to be in an operon with a signal transducing kinase, i.e. they are orphan response regulators^20^. Hence, the environmental conditions to which these regulators respond is not known. The up-regulation of GGDEF domain containing proteins suggest that intracellular levels of cyclic-di-GMP are higher in cellulose-grown cells compared to their glucose-grown counterparts. To test this, we extracted cyclic-di-GMP from both glucose-grown and cellulose-grown cells and quantified this by liquid chromatography^24^. Indeed, intracellular cyclic-di-GMP levels were approximately two and a half times higher in cellulose-grown cells (Fig. 1F). This is the first reported confirmation of a positive correlation between intracellular cyclic-di-GMP levels and cellulose degradation. Our results therefore suggest that cyclic-di-GMP may play a role in mediating catabolite repression and subsequently facilitates the expression of proteins involved in the degradation and utilisation of lignocellulose by *F. succinogenes* S85.

## Discussion

**Figure 2:**
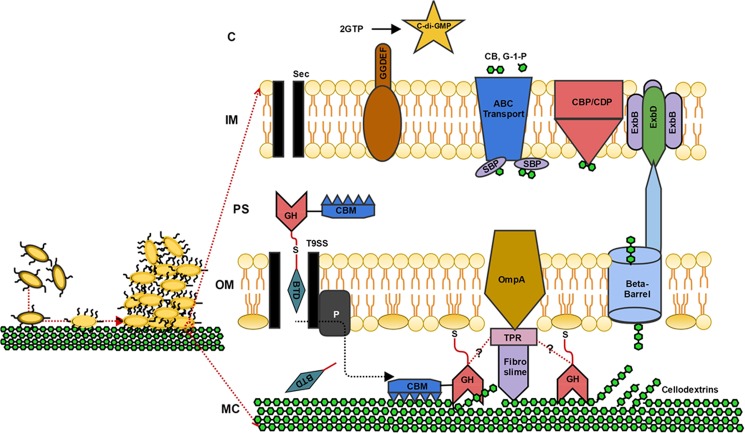
Proposed model of the mechanism of cellulose metabolism in *F. succinogenes* S85. Bacterial attachement to MC cellulose is the first step during lignocellulose degradation. Translocation of cellulases to the surface occurs using T9ss-dependent pathway. On the cell surface, cellulases are attached to Lipid A at the outer membrane via serine residues and form a multi-protein complex along with OmpA, TPR and fibroslime proteins. Released products of cellulolysis (cellodextrins) are then imported to the periplasm via beta barrel proteins and the TonB/ExbB/ExbD active transport system. Cellodextrin phosphorylases cleave cellodextrin into glucose-1-phosphate and cellobiose at the periplasmic side of cytoplasmic membrane, which are then transported to the cytoplasm via the concerted action of solute binding proteins and ABC transporters. **CBM;** carbohydrate binding modules, **GH;** glycosyl hydrolase (figure depicts two GHs as a representative example of the presence of GHs in the multiprotein complex), **BTD**; basic terminal domain, **OmpA;** outer membrane protein A, **TPR;** tetratricopeptide repeats, **CBP/CDP**; cellobiose/cellodextrin phosphorylase, **SBP;** solute binding proteins, **CB;** cellobiose, **G-1-P**; glucose-1-phosphate, **GGDEF;** diguanylate cyclase domain, **GTP**; guanosine triphosphate, **C-di-GMP**; cyclic di guanosine monophosphate, **ABC;** ATP-binding cassette transporters, **T9ss;** Type IX secretion system, **ExbB/ExbD;** biopolymer transport proteins, **MC;** microcrystalline cellulose, **OM;** outer membrane, **IM**; inner membrane, **PS**; periplasmic space, **C;** cytoplasm, **SEC**; secretion pathway, **P**; peptidase.

*Fibrobacter succinogenes* S85 is a Gram negative bacterium, isolated from the rumen of herbivores. It is capable of degrading lignocellulosic biomass; but specialises in the utilisation of crystalline cellulose and its degradation products for survival and growth. The genome sequence of *F. succinogenes* S85 reflects the organism’s adaptation for lignocellulose degradation, as it encodes a variety of carbohydrate-degrading enzymes^5^. Although, how these enzymes are organised for synergistic action, how these enzymes are regulated and the key proteins involved in the utilisation of the cellulose degradation products have remained unknown. Several models have been proposed for the mechanism of lignocellulose degradation and utilisation by *F. succinogenes* S85^5^. Previous work has indicated that the enzymes involved in cellulose degradation are localised on the cell surface^12,25,26^ and on the surface of vesicles derived from the outer membrane^7^, and that adhesion of *F. succinogenes* S85 cells^4,12,19,27–29^ and OMVs to cellulose mediates the degradative process^7^. The results of the current study allow us to gain further insight into the mechanisms employed by *F. succinogenes* S85 for cellulose degradation and utilisation. We propose a series of interlinked mechanisms as shown in Fig. 2. Sensing glucose limitation whilst growth with cellulose as the sole carbon source, enhances production of the adhesion proteins and the lignocelluloytic machinery.

We identified the most abundant cell envelope localised cellulases and hemicellulases involved in lignocellulose degradation. The expression of these enzymes is likely to be under catabolite repression in *F. succinogenes* S85^26,30–32^ and therefore their expression is enhanced in the presence of cellulose. The proposed presence of a T9ss signal in the C termini of most of these enzymes suggests the involvement of T9ss in the transport and covalent linkage of these enzymes to the outer membrane, as previously observed in *Porphyromonas gingivalis*. In *P. gingivalis*, T9ss signal containing proteins are transported to the outer membrane, the T9ss signal sequence is cleaved by a peptidase and the mature protein is covalently attached to the outer membrane *via* a serine residue^33^. The covalent attachment of the cellulolytic enzymes to the cell surface ensures that *F. succinogenes* S85 is in close proximity to the cellulose surface during cellulose degradation and is best positioned to obtain maximal access to the cellulose degradation products. The enhanced abundance of certain TPR domain-containing proteins in the cell envelope proteome of cellulose-grown cells, suggests the presence of multi-protein complexes on the cell surface. Multi-protein complexes are known to play a crucial role in cellulose degradation. For instance, the Gram positive *Clostridium thermocellum*, possesses a cellulosome – a multi-protein complex on the bacterial cell surface, which allows synergistic action of a wide variety of degradative enzymes. In addition to the degradative enzymes, the cellulosome contains typical proteins facilitating protein-protein interactions (cohesins, dockerins) and proteins facilitating both attachment of the complex to the peptidoglycan surface and adhesion to cellulose (scaffoldins)^34^. The genes encoding these typical cellulosomal proteins are absent in *F. succinogenes* S85. Instead, the *F. succinogenes* S85 genome encodes TPR domain-containing proteins known to play a role in facilitating protein-protein interactions, in a fashion similar to cohesins and dockerins^7^. In *F. succinogenes* S85, the synergistic action of the up-regulated outer membrane protein A (OmpA), which facilitates attachment to peptidoglycan and fibroslime proteins that facilitate adhesion to cellulose, could replace the need for scaffoldins. Arntzen and colleagues^7^ have previously reported that these TPR domain proteins are part of a complex with some cellulases, fibroslime proteins and OmpA in outer membrane derived vesicles. The recent experimental observations of Arntzen *et al*. support our suggestion^7^. Arntzen and colleagues provided experimental evidence that proteins found to be up-regulated in our results, i.e. cellulases (FSU_2914, FSU_2362, FSU_2303, FSU_1685), TPR domain protein (FSU_2397), fibroslime protein (FSU_2502), and OmpA (FSU_2396), were found to be in a multi-protein complex (complex 2; spots 6, 7, 8 9 in supplementary material in Arntzen *et al*.^7^) in the outer membrane derived vesicles of stationary phase *F. succinogenes* S85 cells. Therefore, in our study, the up-regulation of these proteins observed in the cell envelope of cellulose-grown cells indicates that a potentially cellulolytic multi-protein complex indeed forms on the surface of *F. succinogenes* S85. The enhanced expression of these proteins on the cell envelope of cellulose-grown cells suggests that such multi-protein complexes are present on the outer membrane and play an active role in mediating adhesion to cellulose and subsequent synergistic activity of the enzymes during cellulose degradation. The cellulose degradation products, i.e. cellodextrins, are then transported across the outer membrane into the periplasm using a TonB dependent mechanism. In the periplasm, the cellodextrins are further processed into smaller subunits by the inner membrane localised cellodextrin phosphorylase. The degradation products of cellodextrins, glucose-1-phosphate and cellobiose, are then subsequently transported across the inner membrane and into the cytoplasm using ABC transporters and their cognate solute binding proteins. To accommodate the protein machinery involved in cellulose degradation, the *F. succinogenes* S85 cell envelope undergoes extensive rearrangements. Peptidoglycan turnover is down-regulated, as is the transport and localisation of much of the lipoproteins and the glycosylation of lipid A with O-antigen. The up-regulation of the cellulose-degrading protein machinery and the down-regulation of peptidoglycan and lipid A glycosylation are in agreement with our previous results, that indicated that the cell surface of cellulose-grown *F. succinogenes* S85 is more proteinaceous and has a lower amount of carbohydrates compared to the cell surface of glucose-grown cells^12^. Therefore, our results indicate that the cell envelope of *F. succinogenes* S85 is extensively geared towards cellulose degradation and utilisation as a consequence of sensing the presence of cellulose and the absence of glucose.

Our results provide us with further insight into a potential mechanism that may be employed by *F. succinogenes* S85 to sense glucose limitation. Glucose is a preferred carbon source for most microorganisms, and a wide variety of nucleotide based second messenger molecules have been deployed by microorganisms to derepress the expression of genes involved in catabolism of sugars other than glucose. The increase in intracellular cyclic-di-GMP concentrations in cellulose-grown cells, suggests that catabolite repression is alleviated in *F. succinogenes* S85 using a cyclic-di-GMP dependent mechanism. The observation that in *F. succinogenes* S85, adhesion to cellulose is an absolute requirement for cellulose degradation supports the involvement of cyclic-di-GMP in cellulose degradation as in other Gram negative bacteria, such as *Pseudomonas aeruginosa*, cyclic-di-GMP is involved in enhancing the expression of genes involved in preferential adhesion to surfaces and subsequent biofilm formation^35^. Cyclic-di-GMP is known to regulate a variety of functions such as growth, motility, adhesion and biofilm formation, which enable the bacterium to sense and adapt to environmental changes^36,37^. This is the first time that cyclic-di-GMP is proposed to be involved in cellulose degradation.

In conclusion, we have undertaken the most comprehensive quantitative proteomic study, to date, of the changes in the cell envelope protein profile of *F. succinogenes* S85 in response to growth on cellulose. Our results indicate that *F. succinogenes* S85 utilises a cellulose degradation mechanism that is more efficient and simpler that the elaborate cellulosome produced by *C. thermocellum*. The enzymatic and the non-enzymatic proteins identified in our study has contributed novel parts to the synthetic biology toolbox for the engineering of recombinant organisms capable of lignocellulose degradation and concomitant production of advanced lignocellulosic biofuels.

## Methods

All reagents used in this work were supplied by Sigma-Aldrich (Dorset, UK) with the highest purity available, unless otherwise stated. All solvents were supplied by Fisher Scientific (Loughborough, UK). Pierce^®^ Cell Surface Protein Isolation kit was supplied by Thermo Fisher Scientific (cat number 89881, Rockford, USA). Lipopolysaccharide (LPS) extraction Kit was supplied by iNtRON Biotechnology (cat number 17141, Kyungki-Do, Korea). Pro-Q^®^ Emerald 300 Lipopolysaccharide Gel Stain Kit was supplied by Thermo Fisher Scientific (cat number, P20495, Loughborough, UK). A 4-plex iTRAQ reagent multiplex kit was supplied by SCIEX (P/N 4352135, Redwood, CA, USA). RapiGest^TM^ SF Surfactant was supplied by Waters (cat number 186001861, Milford, MA, USA). Glucose-1-phosphate colorimetric assay kit was supplied by BioVision, Biosciences (cat number K697-100, Cambridge, UK). Chitinase assay kit was supplied by Sigma-Aldrich (cat number CS0980, Dorset, UK). Sequencing Grade Modified Trypsin was supplied by Promega (cat number, V5111, Southampton, UK). *F. succinogenes* S85 (ATCC 19169), was kindly provided by Professor Paul Weimer (US Dairy Forage Research Centre, Madison, Wisconsin, USA).

### Culture conditions and sample preparation

*F. succinogenes* S85 was grown anaerobically to mid-exponential phase (OD_675 nm_ = 0.6–1.00) in 0.3% (w/v) glucose and 0.3% (w/v) microcrystalline cellulose (Sigma Aldrich, 435236) containing medium as previously described^12^. For glucose-grown cells, cells were obtained by centrifugation (8,000 × *g*, 5 min, 4 °C). To obtain cellulose-grown cells, cellulose-bound cells were obtained by centrifugation (500 × g, 1 min, 4 °C) and unbound cells were discarded. The cellulose-bound cells were washed with phosphate buffered saline (PBS; pH 8.0) two times to remove the unbound cells. To detach the cells bound to cellulose, an additional step was performed using 1 g L^−1^ methylcellulose solution in M8 buffer at 38 °C for 30 minutes suggested by Kudo *et al*. and Olsen and Mathiesen^38,39^.

### Cell envelope protein enrichment by biotin-neutravidin affinity purification

The cells were harvested as described above from glucose and cellulose as the sole carbon source. The cells were washed three times with PBS (pH 8.0) and re-suspended in 4 mL PBS buffer and adjusted to 1.3 OD_675 nm_. To extract protein from the cell envelope, biotin-neutravidin affinity purification was performed as previously described by Raut *et al*.^12^. Briefly, cell pellets were obtained by centrifugation and re-suspended in the 4 mL PBS buffer (pH 8.0) containing 1 mM MgCl_2_ and 30 mg EZ-Link^®^Sulfo-NHS-SS-biotin labels and the mixture was incubated at 4 °C for 30 min. In addition to labelling surface-exposed proteins, EZ-linked Sulfo-NHS-SS-biotin can pass through the outer membrane and thus can not only label inner membrane and periplasmic proteins, but also cytoplasmic proteins^12,40^. Excess biotin was then quenched using 4 mL of 500 mM glycine-PBS solution. Biotin labelled cells were pelleted by centrifugation and re-suspended in the 4 mL of radioimmunoprecipitation assay buffer (RIPA) (25 mM Tris-HCl (pH 7.6), 150 mM NaCl, 1% (v/v) NP-40, 1% (v/v) sodium deoxycholate, 0.1% (w/v) sodium dodecylsulphate (SDS)) and protease inhibitor cocktail set II was added before cell lysis.

Cell lysate was obtained by sonication (30 sec sonication, 1 min on ice; 8 cycles). At this stage, oxidised glutathione (100 µM) was added to the lysates to protect disulphide bond in the Sulfo-NHS-SS-biotin. Lysates were centrifuged (16000 × g, 10 min, 4 °C) and the supernatant containing the biotinylated proteins was collected. The clarified lysate was incubated with neutravidin agarose gel slurry on ice for 2 hours with gentle shaking. Unbound proteins were removed using by washing two times with wash buffer A (25 mM Tris-HCl (pH 7.6), 0.65 M NaCl, 0.1% (v/v) NP40), and by washing one time with wash buffer B (25 mM Tris HCl (pH 7.6), 1.15 M NaCl, 0.1% (v/v) NP-40) and subsequently with wash buffer C (25 mM Tris-HCl (pH 7.6), 0.15 M NaCl). Each time, the wash buffer was removed by low speed centrifugation (200 × g, 15–20 sec) and discarded. Finally, biotinylated proteins bound to neutravidin agarose gel were eluted by incubation with 5% (v/v) 2-mercaptoethanol in PBS at 30 °C for 30 min and subsequent centrifugation at 200 × g for 15–20 seconds. The elution step was repeated 3 times.

### Total protein concentration estimation

Proteins were precipitated with 10% (v/v) trichloroacetic acid (TCA) and protein pellets were obtained by centrifugation (18,000 × g, 10 min, 4 °C)^41^. Pelleted proteins were washed with ice-cold acetone and air dried. The protein pellets were re-solubilised in 0.5 M triethylammoniumbicarbonate (TEAB) buffer containing 0.1% (w/v) RapiGest. The total amount of proteins was estimated by Bradford assay method according to the manufacturer’s protocol. Absorbance was recorded at 595 nm and the protein concentration was determined using a bovine serum albumin (BSA) standard.

### In-gel digestion of proteins

Two biological replicates of cell-envelope protein samples (each 30 µg of proteins) from glucose- and cellulose-grown cells, were run on SDS-PAGE and in-gel digested as previously described by Karunakaran *et al*.^42^. Briefly, protein bands were destained with 400 µL of 200 mM TEAB in 40% (v/v) HPLC acetonitrile (ACN). Gel pieces were dried in a vacuum concentrator (Eppendorf, Stevenage, UK) for approximately 5 min at 30 °C. Proteins were reduced using 0.125 mM tris 2-carboxyethyl phosphine hydrochloride (TCEP) by incubating at 60 °C for 1 hour. Alkylation was performed using 0.5 mM methyl methanethiosulfonate (MMTS) at room temperature for 30 min in dark. Gel pieces were washed two times with 400 µL of 50 mM TEAB solution for 15 min and once with 400 µL of 50 mM TEAB in 50% ACN for 15 min. Subsequently gel pieces were dried in a vacuum concentrator for approximately 15–30 min at 30 °C. Proteins were in-gel digested with trypsin at a trypsin/protein ratio of 1:50 (w/w) in 200 µL of 40 mM TEAB buffer in 9% (v/v) HPLC ACN for approximately 16 hours at 37 °C. At this stage, 0.1% (w/v) of RapiGest was added. After digestion, samples were centrifuged briefly at 13,000 × g for 10 sec and supernatant was collected in new Eppendorf tube. Peptides were extracted twice with 100 µL of 5% (v/v) formic acid (FA) solution and once with 50 µL of 100% HPLC ACN. Finally, all the supernatants were combined, vacuum dried and stored at −20 °C until further analysis.

### iTRAQ labelling

iTRAQ 4-plex labelling was performed as previously described^43^ and as shown in Supplementary File 1, Fig. 1. Peptide pellets were re-suspended in 20 µL TEAB buffer and mixed with iTRAQ reagents. Labelling reaction was carried out at room temperature for 2 hours with gentle shaking and the labelled peptides were subsequently pooled. RapiGest was precipitated by acidification using 0.5% (v/v) trifluoroacetic acid (TFA) and the labelled peptides stored at −20 °C until further analysis.

### Hypercarb fractionation

iTRAQ-labelled peptides were re-suspended in 100 µL of Hypercarb buffer A (97% (v/v) HPLC water, 3% (v/v) HPLC ACN, 0.1% (v/v) TFA). Peptides were fractionated using a Hypercarb porous graphitic column; 7 µm particle size, 50 mm length, 2.1 mm diameter and 250 Å pore size, (Thermo Scientific, Waltham, MA, USA) coupled with an UHPLC Ultimate 3000 RS (Dionex, Thermo Fisher Scientific, Hemel Hempstead, UK) at a flow rate of 0.2 mL min^−1^. A 55 min gradient was performed using 2% buffer B (97% (v/v) HPLC ACN, 3% (v/v) HPLC water, 0.1% (v/v) TFA) for 0 min, 2–10% B for 5 min, 10–60% for 30 min, 60–90% B for 1 min, 90% B for 6 min, 90–2% B for 1 min and 2% B for 12 min. Fractionation and chromatography was monitored at the wavelength of 240 nm through Chromeleon software (Thermo Fisher Scientific, Hemel Hempstead, UK). Fractions were collected every 2 min from 10 min to 50 min (20 fractions). Collected fractions were then dried in a vacuum concentrator and stored at −20 °C until further analysis.

### LC MS/MS analysis

Each fraction was re-suspended in 10 µL reverse phase (RP) buffer A (97% (v/v) HPLC water, 3% (v/v) HPLC ACN, 0.1% (v/v) FA) and combined to obtain 4 fractions for mass spectrometric analysis. A Q Exactive^TM^ Hybrid Quadrupole-Orbitrap^TM^mass spectrometer (Thermo Scientific, Bremen, Germany) coupled with an online UHPLC Ultimate 3000 (Dionex, Thermo Fisher Scientific, Hemel Hempstead, UK) was used to analyse the fractions. From each fraction, 5 µL were injected two times into the system, online peptide separation was performed by PepMap RSLC C18 column (2 µm, 100 Å, 75 µm × 50 cm) (Thermo Fisher Scientific, Hemel Hempstead, UK) at a constant flow rate of 300 nL min^−1^. A 135 min gradient was performed using RP buffer B (97% (v/v) HPLC ACN, 3% (v/v) HPLC water, 0.1% (v/v) FA) as follows: 4% B for 0 min, 4% B for 5 min, 4–40% of B for 100 min, 40–90% of B for 1 min, 90% B for 14 min, 90–4% for 1 min and finally 4% of buffer B for 14 min. Mass spectrometry (MS) data was acquired using Xcalibur software v 4.0 (Thermo Scientific, Bremen, Germany) with the following settings. MS scans were acquired with 60,000 resolution, automatic gain control (AGC) target 3e6, maximum injection time (IT) 100 ms. The MS mass range was set to be in the range 100–1500 m/z. Tandem mass spectrometry (MS/MS) scans were acquired using high-energy collision dissociation (HCD), 30,000 resolution, AGC target 5e4, maximum IT 120 ms. In total, 15 MS/MS were acquired per MS scan using normalised collision energy (NCE) of 34% and isolation window of 1.2 m/z.

### Data Interpretation and protein identification

The *F. succinogenes* S85 (taxon ID: 59374) database containing 2871 proteins was downloaded from Uniprot (.fasta) and uploaded on MaxQuant software (version 1.5.4.1). The settings are as follows; For “type the experimental set” MS2 and 4-plex iTRAQ were selected with reporter mass tolerant 0.01 Da. Enzymatic digestion with trypsin was specified and two missed cleavages were allowed per peptide. Oxidation of methionine and deamidation of asparagine and glutamine were selected as variable modification and methylthio modification of cysteine was selected as the fixed modification. The false discovery rate (FDR) at the peptide spectrum match/protein level was set at 1%. The reporter ions intensities (114, 115, 116 and 117) were used for quantification purposes. Isotopic and median corrections were applied using an in-house automated method as described by Ow *et al*.^44^. Fold changes of the differentially abundant proteins were calculated using a method described by Pham *et al*. with 95% significance^45^. Further details regarding identified peptides, quantified proteins and calculated fold changes for the regulated proteins are supplied in two separate excel files (Supplemental Information S1 and S2).

### Lipopolysaccharides (LPS) extraction and analysis

LPS were extracted according to the manufacturer’s protocol. Briefly, three biological replicates were used for both glucose- and cellulose-grown cells. Cells from 5 mL of a mid-log phase culture, corresponding to 5 × 10^8^ cells, were lysed with 1 mL of lysis buffer. Chloroform was added (200 µL) and the mixture was vigorously vortexed for 10–20 sec and incubated at room temperature for 5 min. The supernatant was clarified from the mixture by centrifugation (13,000 × g, 10 min, 4 °C) and collected in a clean Eppendorf tube. Purification buffer provided by the manufacturer was added (800 µL) to the supernatant, vortexed and LPS pellets were obtained by centrifugation (13,000 × g, 15 min, 4 °C). The extracted LPS were washed two times with 1 mL of 70% (v/v) ethanol and air dried. The extracted LPS were re-dissolved in 70 µL 10 mM Tris-HCl buffer (pH 8.0) and boiled with Laemmli sample buffer for 5 min. SDS-PAGE gel was performed with 12.5% resolving gel containing 4 M urea and 4% stacking gel. The running buffer (2.5 mM Tris-HCl, 19.2 mM glycine, and 0.01% (w/v) SDS, pH 8.3) prescribed by Guard-Petter *et al*.^46^ was used. Gel staining was performed as per the supplier protocol using Pro-Q^®^ Emerald 300 Lipopolysaccharide Gel Stain kit. The gels were imaged using a Biospectrum® 410 imaging system (UVP, Cambridge, UK). The densitometric analysis of the carbohydrates in the LPS was quantitated using ImageJ software.

### Cellobiose/cellodextrin phosphorylase activity assay

Cellobiose/cellodextrin phosphorylase activity of the whole cells was measured using the glucose-1-phosphate (G1P) colorimetric assay kit. Each assay was carried out using three biological and three technical replicates. Briefly, cells from mid-log phase culture corresponding to 1 × 10^8^ cells from glucose and cellulose culture were harvested and washed two times with water under anaerobic conditions. Anaerobic conditions were not maintained in the subsequent steps in which the cells were resuspended in 200 µL of 50 mM sodium phosphate buffer containing 20 mM cellobiose substrate and incubated for 1.5 hours at 37 °C. Supernatant obtained by centrifugation was mixed with 20 µL of stop solution (4 M Tris-HCl, pH 7.0). G1P assay was performed using 50 µL of supernatant and 50 µL of reaction mixture (44 µL, G1P assay buffer; 2 µL G1P enzyme mix; 2 µL G1P developer and 2 µL G1P substrate mix). The blank was prepared using 50 µL supernatant mixed with 50 µL reaction mixture without enzyme mix. Standard curve was obtained by preparation of G1P standards (0 to 8 nmol). Absorbance was recorded at 450 nm.

### Chitinase activity assay

The endo-β-N-acetylglucosaminidase activity was performed using a chitinase assay kit. Each assay was carried out using two biological and two technical replicates. Approximately 1 × 10^8^ cells, were re-suspended in 30 µL of assay buffer and the suspension was used to perform the assay. Ten µL of the suspension were mixed with 90 µL of substrate solution (4-nitrophenyl β-D-N, N′, N″-triacetylchitotriose) and incubated at 37 °C for 2 hours. The reactions were stopped by adding 200 µL of stop solution, provided by the manufacturer, to each well (except standard solution). Supernatants were collected by centrifugation at 8000 x*g* for 5 min and absorbance was measured at 405 nm. 100 µL substrate solution without enzyme was used as a blank. Endo-β-N-acetylglucosaminidase activity in units per millilitre was calculated by applying the following equation.

$$Endo-\beta -N-acetylglucosaminidase\,activity=\frac{({A}_{405}s-{A}_{405}b)\ast 0.05\ast 0.3\ast DF}{{A}_{405}std\ast T\ast {V}_{enz}}$$

*A*_405_*s*; absorbance of sample

*A*_405_*b*; absorbance of blank

DF; dilution factor

*A*_405_*std*; absorbance of standard

T; Incubation time

V_enz_; Volume of enzyme

### *In vitro* quantification of intracellular cyclic di-GMP from *F. succinogenes* S85 by HPLC

Intracellular cyclic-di-GMP (c-di-GMP) was extracted and quantified using two biological and two technical replicates of glucose- and cellulose-grown cells using heat and the ethanol precipitation method as described by Roy *et al*.^24^ with few modifications. Briefly, cell density was adjusted to 1.8 OD_675nm_ in 4 mL and cell pellets were obtained by centrifugation (8000 × g, 5 min, 4 °C). After washing with PBS (pH 8.0), cell suspensions in 400 μL PBS were subjected to heat treatment at 100 °C for 5 min followed by treatment with absolute alcohol. The mixture was subjected to centrifugation and the supernatant containing c-di-GMP was collected. This extraction procedure was repeated three times and the collected supernatants were combined and dried using a vacuum concentrator. Pellets were re-suspended in 25 µL HPLC water and 6 μL were used for HPLC analysis on a Shimadzu HPLC (Buckinghamshire, UK) equipped with a reverse-phase C18 Targa column (2.1 × 40 mm; 5 μm) (The Nest Group, USA). The following buffers were used: buffer A (HPLC water, 10 mM ammonium acetate) and buffer B (HPLC methanol, 10 mM ammonium acetate) and the following gradient was applied: 0 to 9 min, 1% B; 9 to 14 min, 15% B; 14 to 19 min, 25% B; 19 to 26 min, 90% B; 26 to 30 min, 90% B; 30 to 31, 1% B; 31 to 40 min 1% B. Samples were run at flow rate of 0.2 mL min^−1^ and c-di-GMP was detected at 253 nm. Commercially available c-di-GMP was used as a standard and a calibration curve was generated for quantification. Simultaneously, cell pellets obtained after extraction were used for protein quantification. Pellets were re-suspended in 50 µL of TE Buffer (10 mM Tris-HCl pH 8.0, 1 mM EDTA) and lysed by brief sonication. Protein quantification was carried out by the Bradford assay with BSA as a standard. Quantified c-di-GMP was normalised by protein concentration.

### Scanning electron microscopy (SEM)

Cells grown with glucose or cellulose as the sole carbon source were fixed in 2% (v/v) glutaraldehyde in Sorenson’s buffer and fixing agent was removed by rinsing with Sorenson’s buffer. Cells were dehydrated through consecutive washes with 5%, 50%, 75% (v/v) and absolute ethanol. The cells were subjected to critical point dehydration in carbon dioxide using a Bal-tech critical point dryer (Polaron, Agar scientific, Essex, UK). Cells were mounted on a stub with a carbon disc, dried overnight and coated with gold using a SEM coating unit (Polaron, Agar scientific, Essex, UK) (15 nm as standard). The images were inspected using Inspect F FEG SEM (FEI, Netherlands).

## Supplementary information

Supplementary File 1

Supplementary File 2

Supplementary File 3
