# Publisher Correction: Deciphering the unique cellulose degradation mechanism of the ruminal bacterium *Fibrobacter succinogenes* S85

**DOI:** 10.1038/s41598-019-55971-5

**Published:** 2019-12-27

**Authors:** Mahendra P. Raut, Narciso Couto, Esther Karunakaran, Catherine A. Biggs, Phillip C. Wright

**Affiliations:** 1 The ChELSI Institute, Department of Chemical and Biological Engineering, University of Sheffield, Mappin Street, Sheffield, S1 3JD UK; 2 Centre for Applied Pharmacokinetic Research, University of Manchester, Stopford Building, Oxford Road, Manchester, M13 9PT UK; 3 School of Engineering, Faculty of Science, Agriculture & Engineering, Newcastle University, Newcastle upon Tyne, NE1 7RU UK

Correction to: *Scientific Reports* 10.1038/s41598-019-52675-8, published online 12 November 2019

The original version of this Article contained an error where the Figure Legends for Figures 1 and 2 were inadvertently inserted into the main body of the Article. This has now been corrected in both the HTML and PDF versions of the Article.

