# Supplementary material for: Deciphering the unique cellulose degradation mechanism of the ruminal bacterium Fibrobacter succinogenes S85
Source: Sci Rep. 2019 Nov 12;9:16542. doi: 10.1038/s41598-019-52675-8 (PMC6851124; doi:10.1038/s41598-019-52675-8)
Supplement: Supplementary file 1 [file 41598_2019_52675_MOESM1_ESM.docx]

**Supplementary Information**

**Deciphering the unique cellulose degradation mechanism of the ruminal bacterium *Fibrobacter succinogenes* S85**

Mahendra P. Raut^1^, Narciso Couto^1,2^, Esther Karunakaran^1^, Catherine A. Biggs^3^ and Phillip C. Wright^3*^

^1^The ChELSI Institute, Department of Chemical and Biological Engineering, University of Sheffield, Mappin Street, Sheffield, S1 3JD, UK.

^2^ Centre for Applied Pharmacokinetic Research, University of Manchester, Stopford Building, Oxford Road, Manchester, M13 9PT, UK.

^3^ School of Engineering, Faculty of Science, Agriculture & Engineering, Newcastle University, Newcastle upon Tyne, NE1 7RU, UK.

*e-mail: phillip.wright@newcastle.ac.uk


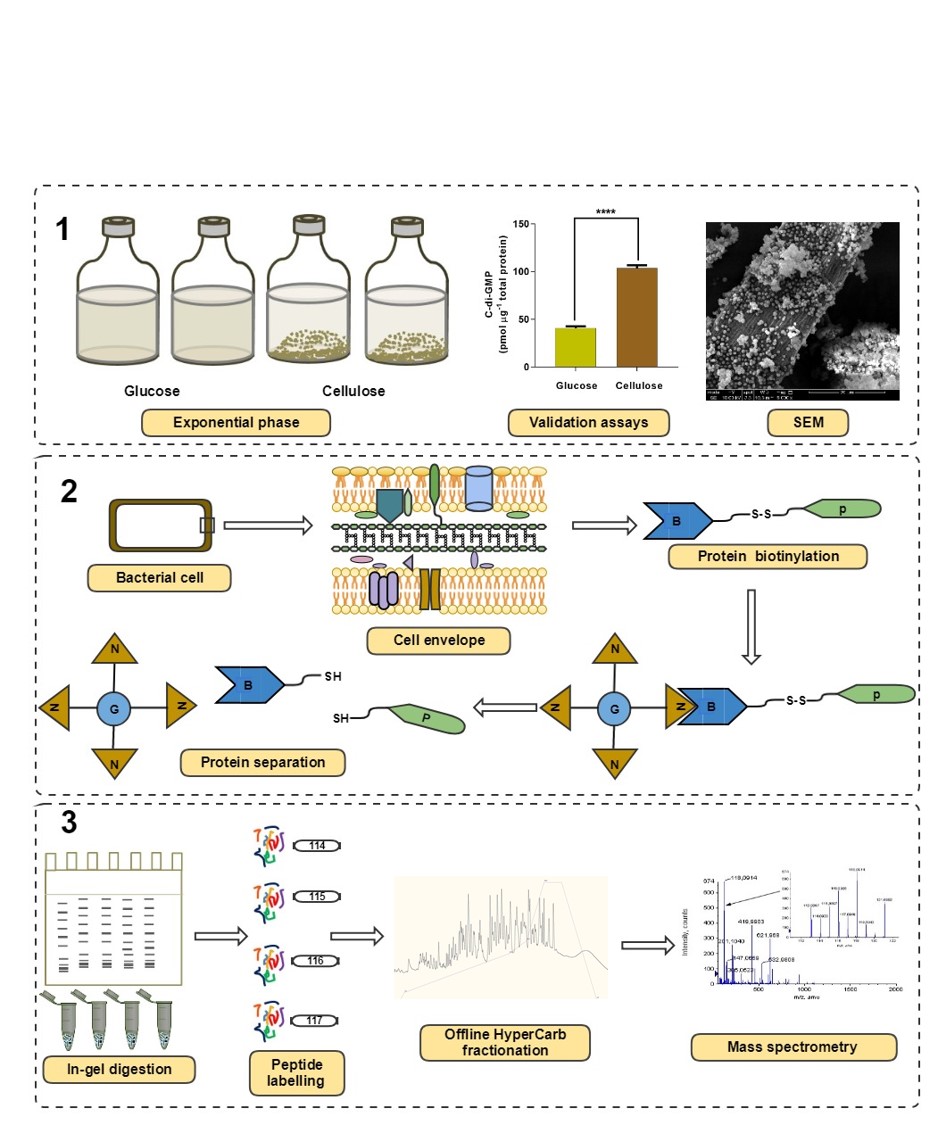


**Supplementary Fig. 1: Experimental workflow. 1)** Experimental conditions, sample collection, validation assays and SEM imaging. **2)** Schematic description of experimental steps in biotin-neutravidin affinity enrichment of cell envelope proteins. Cells obtained from 4 individual samples (2 each for cellulose and glucose) were labelled with EZ-Linked Sulfo-NHS-SS-biotin followed by neutravidin-biotin affinity enrichment and elution of cell envelope proteins. **3)** Purified proteins were quantified and in-gel digested. Peptides were labelled with 4-plex iTRAQ reagents (114, 115, 116, 117) and fractionated by Hypercarb. Protein identification and quantification was carried out as described in the online methods section. **SS;** disulphide bond (SS linker) **N;** neutravidin, **SH;** thiol group, **B;** biotin, **G;** agarose gel and **P;** protein.


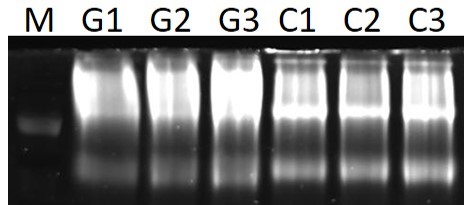


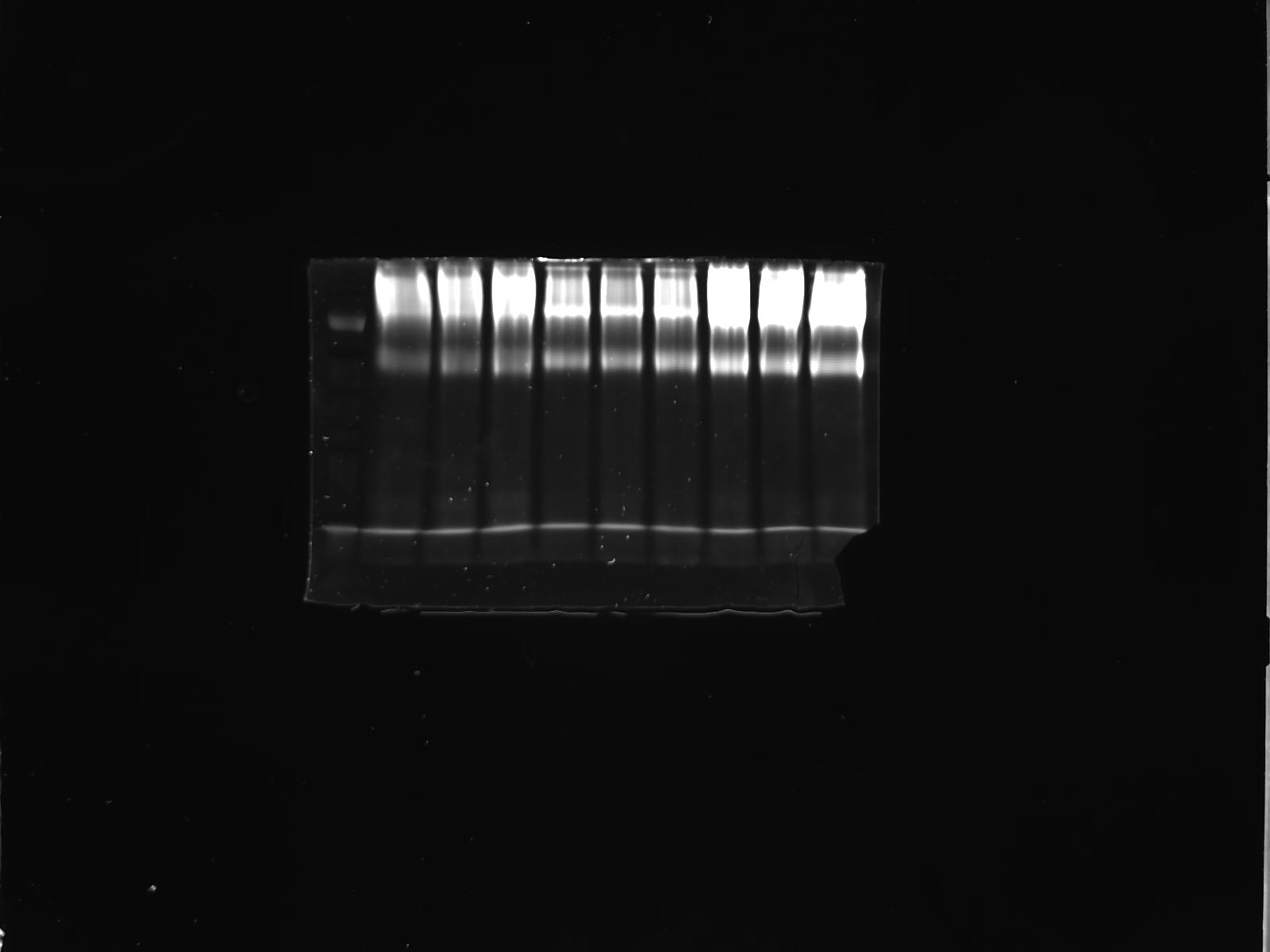


**Supplementary Fig. 2:** LPS samples derived from cellulose (C1, C2 and C3) and glucose (G1, G2, and G3) grown *F. succinogenes* cells. LPS were separated on 12.5 % SDS-polyacrylamide gel electrophoresis and stained with Pro-Q Emerald 300. **M**; LPS standard.

**Supplementary Note:** Differentially regulated GH family proteins bearing a T9ss signal domain (identified peptides from each GH family protein are highlighted in yellow and the proposed T9SS sequence at C-terminal is highlighted in blue).

>tr|A7UG69|A7UG69_FIBSS Cellulase OS=Fibrobacter succinogenes (strain ATCC 19169 / S85) GN=FSU_2914 PE=4 SV=1

MFMKKVFALLTCAAVTSAMAVTASRVGPVSTYGELVANGGKLSGSCPEYSQKAVQVKGMS

LFWSSGNTYSTDFYSEKGINRLVDDMGIEVVRFALGAADEKFNSSGRSYTTGGEGFQKAL

LKSVVNAAIDKDIYVIIDWHIESSDGFTSDAVKFFEYAAQEYGQYNNVIFEIWNEPTGSM

EAVKQHADQVIPVIRKYSDNLILVGSPGWSSQPNACASAGINDKNYGCTLHFYAATHYMG

DGGYNKAAETAMAAGVPVFATEWGTVNANGDGQPDEGSSNKWVEWMAQKGVSWTNWNASA

MNETSAAFANAVFENGFTYTNSGKYVKGKLGGASYKDCGLQNGSASEESGFSAGVANGAT

TSILDDMEDGDRYGYLGGAWAAVEDQENGGASSISNEKIEDDFGNTTYKVVYPVSGDTKN

TSKYVAALKDVKIGKGSLTYGPYIKMFLTLLKEPAKDSPKAYADFSKCKTIKYKYKGASH

NFAIETTDVTDYNYHRVNKDASEGWKEVEITTDMLKQETWGDDSRSKPIKMENATRLSWE

IKGLEKVPDDMNQPKYPYLYVDDVKCDGLSFTAVSGGASENPKSSSSAAVGQSSSSVVAG

SSSSAKATSSSSAIVPGSSATVTYKTVVDIDDVEDLDEVLKTKGTWYAYTDKEPGGKSSI

SNVYDQKLGGYVVAFPGTEDPTNGTKGFVGLKDINWDQGTYTEAPFVALGLNTNADTSKG

IDLSKCGAISYRYKGSAHTFKVQDGSVTDYAYHEYPLDDSQVWKTMVINIEDIAQPNWTQ

DPKDLNWGAIKKMAWEVIGYKGIVYQPTINYLYVDDLKCVevpkvgfktvaraasgikvg

fkgnmlnvnfakageariqvfdmmghvvdsrvanvsaganqfslknmangnyvvrvmmng

aaktarisik

>tr|D9S4N9|D9S4N9_FIBSS Endoglucanase OS=Fibrobacter succinogenes (strain ATCC 19169 / S85) GN=FSU_2362 PE=3 SV=1

MFVKNHALKSVALAALVAAPAFAATAYINQIGYRPGDFKELALVDANGSVDFVNAAGQVV

LSVTPKAASYWDASGQNVQLVDFSKLAEAGKYSIKVNGNVLRSDLVVKSQTYEDIVKASI

KWFYYQRASMALESQYAGKWARAAGHTNPTAELHNSTGASGTINSSKGWYDAGDYGRYIV

NSGITTYTLLSLYEHFPQYFKTLKWNIPAEGSLPDLLAEIKYNLDWMLTMQASDGGVYHK

LTSLGFPGDVMPAADNSKLYAIGKSTAGTFDFAAVMAMASRIYKPFDATYASKCLEAAKK

AYAWGQQNPSRNYLANPSDVSTGAYENDNPNDEKVLAGTELFITTGDASYKQSGSSEYVS

YWGDVMGLATYEKATHQAQFGGDANEAKQKILGTADNFANRAEKGFGVVMAKDDFVWGSN

AVASNQGVWLLHAYYLTGEQKYYKAAVKVLDYLLGKNPLDMSFVTGYGSKSPKMPHHRPS

TSDNIEEPIPGMLVGGPQPGGEDVGSAAEWKCADYRTGQAATAYTDQHCSYATNEVAINW

NAPLAYLAGAIEAINSGYAPEFAAAGVAKQDVPASSSSEAPASSSSSVPQSSSSEVASSS

SAISGNSSSSETVASSSSSQGTIAIHAPVESRMMRSTQPRLRFDDQKVFIEKNGKRFDLK

GHRIK

>tr|A7UG67|A7UG67_FIBSS Endoglucanase OS=Fibrobacter succinogenes (strain ATCC 19169 / S85) GN=FSU_2361 PE=3 SV=1

MKLNFGLKQYIPLAATVLSLATVANAATAYINQIGYRPADPKEFALVDGSGDIEIVNAAG

QTVLQVTPKASSYWAPSTQNVQLVDFTALTVPGTYSIKQGGQVLRSDLKIADKTFEDVTK

AALKWYYYQRASMELEEQYAGQWKRAAGHTNSTVTLHNSTGTSGTIQSSKGWYDAGDYGR

YIVNSGITTYTLLSLYEHFPEYFNTLKWNIPAEGTLPDLLAEIKYNLDWMLTMQAADGGV

YHKLSTLQFPGDVMPAKDTEKLYVIGKGTAASFDFAGVMATTYRVFKTFDATYAAQCLEA

AKKAYAWGLQNPNKAFTNPSDVATGSYSDGELSDEKAFASMELFISTGDASYKPTIDPNK

MSIVPAWPEMYGLAVYAAATHATEVGADAETAKQMLLQYANEFAYAASSGFGVVMSNEDF

VWGSNAVAGNQGVFLLYAYYVTGEQKYYEAAKKVIDYLLGKNPLDMSFLTGFGTKSPKLP

HHRPSTADKITDPVPGMIVGGPQPGGEDIGSKSWECKDYRTGVPATSYTDNRCSYATNEV

AINWNAPFAYIAGALEALNAGYAPSFAAPGVAKGGTSAIKPVVSRNRGKVEHAPRLRFDD

QKVFIEKSGKRFDLKGNHLK

>tr|A7UG68|A7UG68_FIBSS Glycoside hydrolase family 8 OS=Fibrobacter succinogenes (strain ATCC 19169 / S85) GN=FSU_2303 PE=4 SV=1

MIFDMLSNYLLGWMGEIFFLKGIHMKNLAKVMFGVAAVAAVTASAGQFPFPQNMKYPHGK

IIEYADTDMIKDHYKLWKQAWYQASNGWVLAPEGTCSTVSEAIAYGMLISVYMDDQDVFK

KLYNTWTSNSAGANGGMNWRIGCSGGTGTASDADFDAALALVMASKQWNDASYLSAGKSL

ISWIASNDIASNKIKPGNQWNDGFNPSYATTANFQLFQDVAGGSWSSVISQAYTDLNACQ

DSKTGLVPDWCDWNSHKPILTSAAVSNDIGFYDDAARTPWRMAMAYYWYGDTKAQAFNKK

VVSWLIPETRTASGVNSGYKYEGGAYHIDNSDIRRFVSSTFSGGLGLATSSIDSKEAETY

LGTVYKVLKEKKSCSTAQGCGEGSVEGEKYYPATLNMIYLLLVTGNMPNLYNTTGFTPFT

PDPSKAPSISEGEGTHLEFGDTTVSVSGLWNWGAYHDKLGIGTKMVPDSGASPLYRLDDG

SIVARASMEIGPEPEWTEAAAKAGTLKYPSAGIAVSFKKDDCKKDKSCGVNFKTLGIQYI

RVTAKTSGPIRMAILNTITDENEEKKVENAGAGSEPGIYVDNSEEFKAVTYDMTPYEYGF

KGLGDGKEINILDWVSKNNAPEGGEILTCIKGLKWEVKDAKGGLGELTISAVEFLDASKQ

AVDPVKLTGMEIKGPTIGLYKVTFAPSFSVRADGMKLQISGAKAGNVFMVYNMQGKAIAG

GMLMNSNLTVNVPSAGSYIVRVGSEMNRVNVK

>tr|C9RQJ1|C9RQJ1_FIBSS Putative cellulase OS=Fibrobacter succinogenes (strain ATCC 19169 / S85) GN=FSU_1685 PE=4 SV=1

MKLTKLLTSILAGAAISAFAADASTATPKKVGPVSYYGALHTSGSKIIGAKNNQQVMLRG

VSLFWSDATGLSYYNPTVISWVVDNLKIDVFRYAMGIEYYDSNGGTKNKLDDQVSYTKSP

EGQLSTIDRMVQAAIENDVYIVIDWHSHRAHLETNLAKDFFAKISQKYKDVPNIIYEIYN

EPVSGSGGDWGAIKNYANSVVPAIRTNTQNLIIVGTPNWSQHPEQGARDPIQSTNIAYVL

HFYASSHSKGSFGGYVSSALSAGYPVFISEWGTTNADGDGEPNSSATNEWTQFMDQNMIP

NCNWSLRQQTSDVDQKSEKSAIFAGDKSLITAAALDAATFTSSGNIIKSYLTKNARSWAD

SLVKGKSGSCSFKATTAKQTDGKISGVLKSGCTYTSSNEKVASASGSDIVVGDYGFAILT

GNDGSQSIVTVKQVAGQTITNLSDITCNFSGSCTAASKNGMSRDFDGDGTKDYLLTMDDK

TNEGSKFTLTSLDPTTVSVSKNKCTNANCSNSQKNQQVWMLHFHKYGTAKVVASAPAITG

FRAMQDTFEITYKKGSYNMNNKFKDQTIALGGTTTEGFPDALLGNAVTYTFNGQPTTPYL

TKVGNAFVGGAQNAIFAITAHIAETADYEEFERTVTVIVGDAGTAVNLAEWIAYTNPIDA

IKPANKIVNSFNARMDGSMLQFTTKNTGLVKVDIYDALGASVKQMSEVYGKGSHAIDLKG

LPNGSYTLVVRQGSQKASIRWVNK

>tr|C9RMD2|C9RMD2_FIBSS Putative endo-1,4-beta-glucanase OS=Fibrobacter succinogenes (strain ATCC 19169 / S85) GN=FSU_3149 PE=4 SV=1

MNRLVKMGLAGLFAFGLAQAKVNFPFPQMSDYGGNATLLSDKAKASEELKSQFAYWLKTM

YNENGDVAGVRSDPGSDTYFSEGVGYGMLLMVYFSDNTTSYQPQFDKIWNFYKKMMNENG

LMIWKVGNLSQSWDAGNGAALDGDIDAAAALAMAYFQFGDEKYKEDAKKLIQAMKKSEFE

SNGLHLPGDKWGDAALNRKNPGYFDPAYMPVFALIDTDNAEFWNKTAYDANMKLYEASSA

EVTTGLIDDWTDKNGKSEDDYYSYDASRAPWRNAKAVCWHGDTRALALDKKMAEFVSTVS

AANMKGPVLRSSGSLGNDHNSTFVTSLMTALISDAKYQAKLDEYWKEAVALGDENYFNQS

LKLLNGLLVSGNMPNLAAATPSQPTSSSSVTPESSSSDATIALPTLATKSPKMTLSGRSL

QITANGNVHVDLISMTGSVLKSFDNHANGSLNVSLKSVPNGLYVVRVKNAGVTSLKKIKL

D

>sp|P35811|XYNC_FIBSS Endo-1,4-beta-xylanase C OS=Fibrobacter succinogenes (strain ATCC 19169 / S85) GN=xynC PE=1 SV=2

MKTFSVTKSSVVFAMALGMASTAFAQDFCSNAQHSGQKVTITSNQTGKIGDIGYELWDEN

GHGGSATFYSDGSMDCNITGAKDYLCRAGLSLGSNKTYKELGGDMIAEFKLVKSGAQNVG

YSYIGIYGWMEGVSGTPSQLVEYYVIDNTLANDMPGSWIGNERKGTITVDGGTYTVYRNT

RTGPAIKNSGNVTFYQYFSVRTSPRDCGTINISEHMRQWEKMGLTMGKLYEAKVLGEAGN

VNGEVRGGHMDFPHAKVYVKNGSDPVSSSSVKSSSSTDAPKSSSSKGNGNVSGKIDACKD

VMGHEGKETRTQGQNNSSVTGNVGSSPYHYEIWYQGGNNSMTFYDNGTYKASWNGTNDFL

ARVGFKYDEKHTYEELGPIDAYYKWSKQGSAGGYNYIGIYGWTVDPLVEYYIVDDWFNKP

GANLLGQRKGEFTVDGDTYEIWQNTRVQQPSIKGTQTFPQYFSVRKSARSCGHIDITAHM

KKWEELGMKMGKMYEAKVLVEAGGGSGSFDVTYFKMTDKAHPLAQPEPESSSSEAKVESS

SSTVALHAAPKMELKSGNFQVFDMQGRFLGTVKLDAGASVAQVLKANFKNAGIYMVKQGN

FMQRVAVK

>tr|C9RS20|C9RS20_FIBSS Putative endo-1,4-beta-xylanase OS=Fibrobacter succinogenes (strain ATCC 19169 / S85) GN=FSU_2263 PE=4 SV=1

MKLLKKVTMFGLLAGFGVSALADNPISTYHYLADPGAAADDDYFYIITDSDDPAPANSNG

YKIYALYAFRSRDMQNWTDYGIIYDARKVSGINDIWASGIAVHNGTFYIVFPDGGGGGIG

YIKAPAIEGPWTNAVGQGKDKLVGGRGIIGCDGVSWCFDPGIFIDDDGTTYVTWGGGEST

SRPNTDNFDIVKLNDAKNAPVGNGSHVKVNNLPTRKMLEASYIHKHKGTYYFSYSTGWQQ

GAPTIDYGTSNNVMGPYTWKGTILGDPSMNGRSINGNNNHHGIAEFKGHSYVVYHDRRIA

KGHNGLEIIPADDGQPKPNEGYHRSVSVDEMFYNADGTIQTVKVTDEGPAQIENFDPYDW

YPALTSSKQKGIRSRSNFVQGKAAEHVLLPLSSKESWLRVSGVDFGTAATGFTVEAASAA

DNNKIEIRTGSATGTLAGTCTLKNTGNKNTYAENKCEVEGLKGIVKQLFLVFKGDRDSTM

AIKAWGFEGSGTTPPEPQKPFSGKAWEIPGKIEMEDFDIPGSGRGSEIKSYSENDSEDHG

IENGGKSYREDTGVDIYKKATGYVVGYNQSGEWLEYTVNVKEAGDYTMFASVATDNSTAS

FTLSIDDKSIAEVPVSGDSWDDFVKVKANVTLPAGEHVLRFTVTGDWFDIDYMTFAKGKD

AKDPDDETIGIKGFRVLGADAVANFDVFDLTGKKVSSFTARNIHEAKKLWRENPQSRNVQ

GVCIIRNRYNGAVARVRTTR

>tr|C9RS21|C9RS21_FIBSS Putative endo-1,4-beta-xylanase OS=Fibrobacter succinogenes (strain ATCC 19169 / S85) GN=FSU_2264 PE=4 SV=1

MRLLKRLTMAGLLAGFGVGALADNPISAYHYLADPGAAADDTYFYVITDSDDPAASNANG

YNIKALYGFRTKDMKNWTDFGIIYDARKVDGIGDIWASGIAVNPNDHRLYIVFPDGGGGG

IGLIGADSIAGPWTNPVSGNKKLINNWGGGLADCDGIGWCFDPAIFFDDDGQGYFTFGGG

ESNSRPAANNNNNIFNIYKLNKDMKGFDVGSKTQLKIGGPKAMEASYIHKYKGNYYLSYS

TADLRIAYGMSKNPMGPYEYKGIFMGNPNINGQNINANNNNHHGIAEFKGHWYVAYHDRR

IANGYDGLEKIPADDGKANPVPAFHRSVSVDEFFYNGDGTMKELTFTKEGPKQIENFDPY

DWYPALTSSKQKGIRSRSNWTPGKVAEHLLLPLSTKESWIRVSGVDFGTAATGFVVQAAS

AADGNKIEIHSGSATGTLAGTCTLKNTGNKNTFAENSCEVTGLKGIVDEIFLVFKGSQDS

TMAIKAWGFEGSGTTPPEPQKPYGEKAVTLPAKIEAEHYDIPGVGRGGDVDSYSDNEKAN

QGDAKFREDQGVDIVEGGTGMAIGYTASGEWLEYTVEVPEDGDYAIKASASTGMESASFC

FLADGKAIGDTITVPQTGEDWSVYKEFEGGKAKLTKGTHVIRLVITGDNVNVDWFSLGEV

SEVGLKPAVKFQANASRVYRVYSVSGKLLGTVELVGKKAAEALQSAGFNKGVYMLKSVDG

HKTFMTSVAR

>tr|C9RS26|C9RS26_FIBSS Xylanase/xylosidase OS=Fibrobacter succinogenes (strain ATCC 19169 / S85) GN=FSU_2269 PE=4 SV=1

MGLFSKMAKSAVVLAAFAVVNSAAVKVNNPIMYVDSPDPSIVRVDDAYYMVTTTMHFAPG

VPVFKSTDLAQWRTVGYAYQTLTNNDQQNLNGGKDAYGKGSWASSIRYHKGFFYVLTPSY

TTGKTHLYKTADVESGQWSEVQLPFYHDPSLFFDDDGTVWVFYGSGDQISYVQLNDDASG

VKAGGKSGKLGGVSVNQVTGTSNYYVQQEGSHMEKVNGEYYLFTISWPAGKSRSEIVYRS

KSLLSGFSGRYFLSDNGVAQGGIFDTPDGKWYALLFRDSGPVGRMSHLVPMEWKDGWPVP

TSGSKAPSTIDLPESPLPGYGMVTSDDFESGELALEWQFNHNPDNKNWSLSANPGFFRIT

TGRTDSRVVNAKNTLTQRSFGPKSSGRTLVDGKGMKDGDMAGLVALQDDKGFVALAKDGG

NYKVVMYSGNKDGERLVTSENLSDSKVYLRIDFDLPIDRGTAYFYYSTDGSSWKKIGNDV

KLNYDLHMFVGVRWGLFNFATKQAGGYADFDWFKVGTDVNDEIYLDGAGSEPVPQTPFCA

AGENCPANAIPGKIEAEDFDVPGKGKDGSSYYDSDSENHGDSDYRKGTGVDLYKKATGVI

VGYNSEGDWLEYTVNVKEAGDYTMFAAVAAAGSTSSFKLSLDGKDITEELSVPAASSGEE

NYDDYNKVKANVKLPEGEHVLRFTVVGSWLDIDYFTFVKGANATDPDPIVGLAKGVRYNV

QGVQTYGVYGLNGKFIGRVDASNNFDVRSKVNSLVKESGVYIVKSLTTGNTHRLSVTK

>tr|D9S442|D9S442_FIBSS Xylanase/xylosidase OS=Fibrobacter succinogenes (strain ATCC 19169 / S85) GN=FSU_2274 PE=4 SV=1

MKKTSKILLAFGLGFASNALAENPIIQTYYSPDPAPVVFGDTVCVYTGNDEGGSFFTMHG

WRVSCTTDMVNWTDMGELILTNESFGGNAKKNGDWAAQVVRRNGKYYYYVTVESTRGGRA

INVAVADKPEGPFKDARNGQHLAGPNWDYIDPTVWIDDDGQAWLYWGNPKLYYAKLKENM

IEFDGDIKVTDMSRGFSPSGNSVYTEGPWIHKRDKKYYMIYASHGVPEKISYSTSDSPTG

PWKWGGVIMDQGNGTAFTNHSGLIDFKGRSFFFYHNQKNVSGGGYSRSTAVEEFTWNADG

TIPTIKSTNDGVKKPIKNLDPFTRVEAETKSWVGGINVDKSGGYTIIKHVAKQGDNVYLT

NMGSNFYTKVRSVDMGDGADRIIVCTRGNGGKIELHAKSETGATLATMNIPASSSWQENT

FDLKDAAGVEDLFFVVKQGGFDFDYWYMESEKTAVPQTPFKEVASAIPGKIEAEDYDVGG

HNKAFYDNDRENKGGAYREDEVDIVQIDSADKSKGFALGYTEDGEWVEYTIDNQIASEYT

VRLNMATASDDVGVQFFIDDKEITDVIKAEKGEDWDHYSTVEAKTKEIPKGEHVLRMQIV

GNFVNVDWFKFCMGFDCEESSIALPKSRVELQIPEKIYAVFGMTGKFLGNVEVNGQSVAK

SIRAAGFTPGVYMVRSVGQSKTFRVLVK

>tr|C9RS45|C9RS45_FIBSS Beta-galactosidase OS=Fibrobacter succinogenes (strain ATCC 19169 / S85) GN=FSU_2288 PE=3 SV=1

MNFGSSLSLVLSAGLLSAVSLFAQPNDEWNGKPRIFGVNRLNPHVTSMPYTTVEEAVKGD

RHASEWYQTLSGEWRFYHVDKPSQRNNDFYKDNYDVSKWDKIKVPSSWQLLGYDHPIYTN

VIYPWSQNNRVSAPYAPTDFNPVGHYRRTFTVPETWDGKRIRLHFEGVESAYYVWVNGNY

VGYSEDTFTGHEFDINKYLRKGENNISVQVFRWCDGSWLEDQDFIRLSGIMRDVYIYAVP

QVHIQDFQIDATLTNNYKDGLLKTTAWIYNSTGKQSGDYTVELSLYDASGAEVIKPTSQK

VSGIGPNGAEKSVHFEIPYSSPKRWSAETPDLYTAVLTFKDADGKILQVESNKIGFRKIE

IKKDNGAPRLYVNGMPVKFHGVDRHELDPDNGRAVTYDRMEKDVILMKRFNINALRMSHY

PNNPVMYDLCDKYGIYVIDEANVESHGANNDLPKNSDDWRAPAVDRMNSMVQRDKNHPSI

ILWSLGNEAGNGNVFASERQRAHEIDSTRFVHYEGDWNNADVNSWMYFGPDAIQNYRDAN

KPIMLCEYEHAMGNSVGDLQEYMDAFYGNPRSFGGFIWDFIDQGLRHKGTPYFEFGGMWG

DWQNDDNFCANGLVFPDRKIQPEMWEVKYQYSQVRVKNIDAAKGKIQIESRYLYKNLGDF

LDAYWQIRENGKVIKEGKLNGTQMNIGPNEKKEVTIEMPKIETTVGAEYFLDIDFRLKKD

ELWAKAGYSIGHEQFGIDLGQLWSTEIDISTLPTYKVNKSNGLEIEGSDFKIKFDERNGT

LASYVLDGDTIIKNGGIPNFWRAPIDNDKGFNMERGHGEWRKASQKRNVTSEVKEVSQQE

TQVTFNFSFPDVGGTKMKMTYYVYGSGDIVVSYTLNPDGSKSYLPNVGTLFTVPGGYEKV

RWFGRGPDENYIGRNRGSFMGLYSTLADSMTIKYMEIGETGQRTDVKWATLTNEKTGKGL

MIVGNPRMEFSAQHYTPEQLTNVKLPWELKRDKDITLRVDLHQMGVGGINSWGAQPLDAY

LLKANREYSHTFRLAPIRKQLNDPTEYSLLGFKNFGWNKEIPPAKYGLDEINKIYEKQPE

KDITEGANPDTEGLIPVALPGKIRNLAVAEKNYNVFDAQGKKVGAFTTRGVEDLHAITAG

LVKNSGVYIVKAKNGGQAFRITVKK

>tr|Q9F4L0|Q9F4L0_FIBSS Beta-xylanase OS=Fibrobacter succinogenes (strain ATCC 19169 / S85) GN=FSU_2292 PE=3 SV=1

MRKFSLSLAAVAVAGFVSVANAALADGGAKFLGNITTGGQIRSDFAQYWNQITPENGCKW

GSIHSLSNGNSGTSKFAWDNFDKCESAYKWAKEKPGERHFKFHALVWGSQYPNFLCKKKN

PGITVELTKKYITEWFDAVAAKFPDLEYIDVVNEAIWAGNNYHSGYGKPAAGAEGHSTDD

TECGGSYIIEALGGDRVVNGKHQYDFITNAFKMARERWPKAVLIYNDYNTLSWQINEGIE

LIQTIVKNGAPVDAYGQQAHDCKGMSKNDFESKMTRIHNETGLPLLVSEYDIGEADDTKQ

KNDYANQIPFMWETPWVAGITIWGYINGSTWVQNTGLIEKDGRKRASMNWLEDYFAKNLS

KGKNDVTFTPVEPEPQLPFKGEAIAIPGKVEAEDFDIPGVGVNEDGTSNQSYGDDSENHG

DSDYRKDTGVDLYKKATGVIVGYNSEGDWLEWTVNVKEAGDYTMFAAVAAAGSTSSFQLS

LDGKALTEKITVPAAKEGEENYDDYNKVKANVTLPAGKHVLRMDVTGAWFDVDYFTFVKG

KDATDPEPIDVPDAIQSNLRMNYPVLSDYDVFDMNGVRLGRMSAYSVDEAVTTLKNTSAI

KVQGIYLLRSVKNGAVKTVRIAR

>tr|Q9F108|Q9F108_FIBSS Beta-xylanase OS=Fibrobacter succinogenes (strain ATCC 19169 / S85) GN=xynE PE=3 SV=1

MKSKYLKGLLAAALLGGAVSSYAGPGLADGAAKFIGNITQSNSVGSDFTALWNQATAENG

CKWGSVEGTRGRYNWGACDAAYNWAKQNGGHFKFHALVWGSQYPNWLNGLSTDETKKAIT

AWFDAVKEHYPDLEMIDVVNEAIRTGNNSYHSPYGKNNNIIPALGGDNGGNYQFVTTAFK

MARERWPKAILIYNDYNTVQWNKDQGIQLIQTIKKNGAPVDAYGLQAHDMMSQGGGQGGT

GGGGVCLNINTLKSVLKEIWDKTQTPMFISEYDIATTDDNIQKQCYSEQISHFMENEHIA

GITIWGYIYGRTWLDCNGTASGCSGIVKNGQDRAALKWMREYLKSNKGVNTTGLNTGVLT

PVDPVPQEPFKGEALAVPGKIEVEDFDKPGQGKNEDGTSNESYGDDSENHGDSDYRKDTG

ADLYKKATGVALGYNTTGDWYEYTINIAEAGDYTAIASVATEGTGAFTLSLDGKSLAEFE

VTGTSYDDFSDVKKKVTLPAGKHVLRLDVTQQYFDIDYINFVKGEVADNPGGNDNPGGGD

EPGPGVGIKTSVQYQAPRIGSYDVFDANGVRLGRMNAYSMSEAAQILKSSNDVKNNGIYM

LRSVQSGAVKSVRISR

>tr|C9RS30|C9RS30_FIBSS Alpha-galactosidase OS=Fibrobacter succinogenes (strain ATCC 19169 / S85) GN=FSU_2272 PE=3 SV=1

MFGLNKKNGCKLGVLATLTLVGLASQAFASADTLVLTPPLGWNSWNVFHENINEKQIQEI

ADAMVESGLRDAGYVFLNLDDNWMDTKRDAQGNLQNNPKTFPSGMKAIADYVHKKGLKFG

LYGDRGKRTCHHYNSNWQSESGSNGHEVQDAKKLAEWGVDYWKYDNCDSDPRTQEKDYTA

MSNALRNSGRDIVFSICMWEYKDWMPKIANLWRTTFDIGPEWISTSWYRGVYEIIDANNK

YWQIAKPGHWNDPDMLEVGNRGLSYEEQRSQMTMWSIMAAPIMISSDVRNMSNETKELYL

NKDMIAINQDSLGVQGHRISDKNGKQVWTKPLKNGDIAVALLNNNNSTQTVECNFKDIGV

EGEVEVRDAWKKKDLGPVSSVSIELPAHGSALLRLVLKPVPREPFKGKPLDIPGKIEVED

FDVNGVGQGNTTYNESDTENHGDSDYRPGTGVDLYKKATGVIVGYNQAGEWLEYTVNVAK

TGTYTMSASVASANSTSSFKLSMDGKDITEEIAVPAATAGEDNYDEYNTVEAKVSLTEGE

HILRFTVTGDWMDIDWIEFTDGTVGLNKVRLTSFESENSYNVFSATGKHLGRVDLNGASM

PQALKNAGYARGTYMVRSVKGNQIQRVNVR

>tr|C9RLJ5|C9RLJ5_FIBSS Glycosyl hydrolase, family 16 OS=Fibrobacter succinogenes (strain ATCC 19169 / S85) GN=FSU_2986 PE=4 SV=1

MKKNHVIASIVALASIAFAAPPSNFSGWDLVFEDNFDGTSLDKKKWNPTYNWGPTHNHRA

YCAEENVIVSDGTLKLKGEKKMHPDAKGRKAKFNNKEIPVDYTSGAIDTKGKFEVKYGYI

EGRFKAPSQKGTWPAFWTLQDGWPPEIDILEIPASRKQHHYYLHYTDPSWYNSHGSAWDH

EASFGGHKDDNVDRSADFHTYAVEWDESTLSFYFDDKKFASYNRPTEIKQLSAQYIIVNL

AIGGWAGDDIEITSDKPAYFEADWVRVWQAKPAKPDTVRIMSMNFGTCMTRTAEDKLALG

DCSGDNAIATITPLSSTTYRINFGDISLDTPNESTDAGVTMGLYKWNGGGHQKVVMEKQS

GYEGSVVRMKMQHSNMYLRATTDGERVVQSWADDWEWNQMWRLLKSDDEIPTKDPSVGIH

ETARTIAPAYGAKVFRKNGMLYVQFGDRNGGVPNNNGTKLEPRAYDFKGRIIK
